# Numerical Analysis of the Effects of TPMS Topology and Relative Density on Heat Sink Cooling Performance

**DOI:** 10.3390/ma19183992

**Published:** 2026-09-19

**Authors:** Welteji Iticha, Tomasz Stręk

**Affiliations:** Institute of Applied Mechanics, Poznan University of Technology, Jana Pawla II 24, 61-139 Poznan, Poland

**Keywords:** heat transfer, heat sink, cooling, TPMS, porous, auxetic, FEM

## Abstract

The trend toward high power density in compact electronic devices has intensified the demand for enhanced thermal management. Triply periodic minimal surface (TPMS) structures are attractive for heat sink (HS) applications owing to their high surface area, interconnected flow pathways, and compact design. This study presents a computational fluid dynamics investigation of porous TPMS heat sinks with gyroid, diamond, and I-WP topologies using COMSOL Multiphysics version 5.1. The effects of geometry, network morphology (sheet and solid), and relative density (RD = 15%, 30%, and 45%) were evaluated through temperature and velocity fields, pressure drop, average Nusselt number, and Darcy friction factor. The results demonstrate that, relative to the I-WP structure, the gyroid and diamond structures achieved 10.9–12.4% and 2.9–4.7% higher average Nusselt numbers, respectively. Porous gyroid structures reduced the maximum temperature by 2.2 [K] compared with the solid counterpart due to their highly interconnected pore network. At an inlet velocity of 0.3 [m/s], the average Nusselt number increased from 11 to 43 as the relative density increased from 15% to 45%, accompanied by a pressure drop increase from 1.9 to 4 [Pa]. These findings demonstrate that porous gyroid TPMS heat sinks with optimized relative density provide an effective solution for advanced thermal management in electronics.

## 1. Introduction

Rapid advancement in electronic technology has increased the demand for highly efficient thermal management systems to extend the service life of devices across different fields [1,2,3]. This is primarily because modern compact electronic devices generate large amounts of heat within confined spaces, making effective heat dissipation essential. In particular, the power consumption of high-performance computing systems can exceed 200–250 [W]. Every 10 °C increase over the operating temperature (80–85 °C) of high-power devices approximately doubles the probability of electronic component failure [4]. Failure to effectively dissipate this generated heat may lead to a decline in operational efficiency, material degradation, unstable performance, and catastrophic device damage. Accordingly, effective thermal management is essential to control temperature rise and maintain the reliability, stable operation, and service life of electronic systems across both consumer and industrial applications. Hence, heat sinks (HS) play a fundamental role in ensuring safe and reliable operation by dissipating excess thermal energy generated during device operation. The thermal efficiency of an HS is determined by the coupled interaction between conduction within the solid structure, convective airflow conditions (natural or forced), and fluid flow characteristics within the cooling channels [5,6]. The geometric design of the structure also significantly affects thermal fluid behavior by determining flow acceleration, channel constriction, vortex development, and local flow stagnation. Consequently, these factors can impact the thermal gradient, Nusselt number, and fluid flow behavior. Moreover, the extension of an effective heat transfer area controls the development of the thermal boundary layer, intensifies fluid mixing, and consequently reduces convective thermal resistance. Simultaneously, an increase in airflow velocity suppresses the growth of the thermal boundary layer and improves the convective heat transfer coefficient, thereby enhancing the overall heat dissipation capability and enabling the removal of higher heat loads [7].

**Figure 1:**
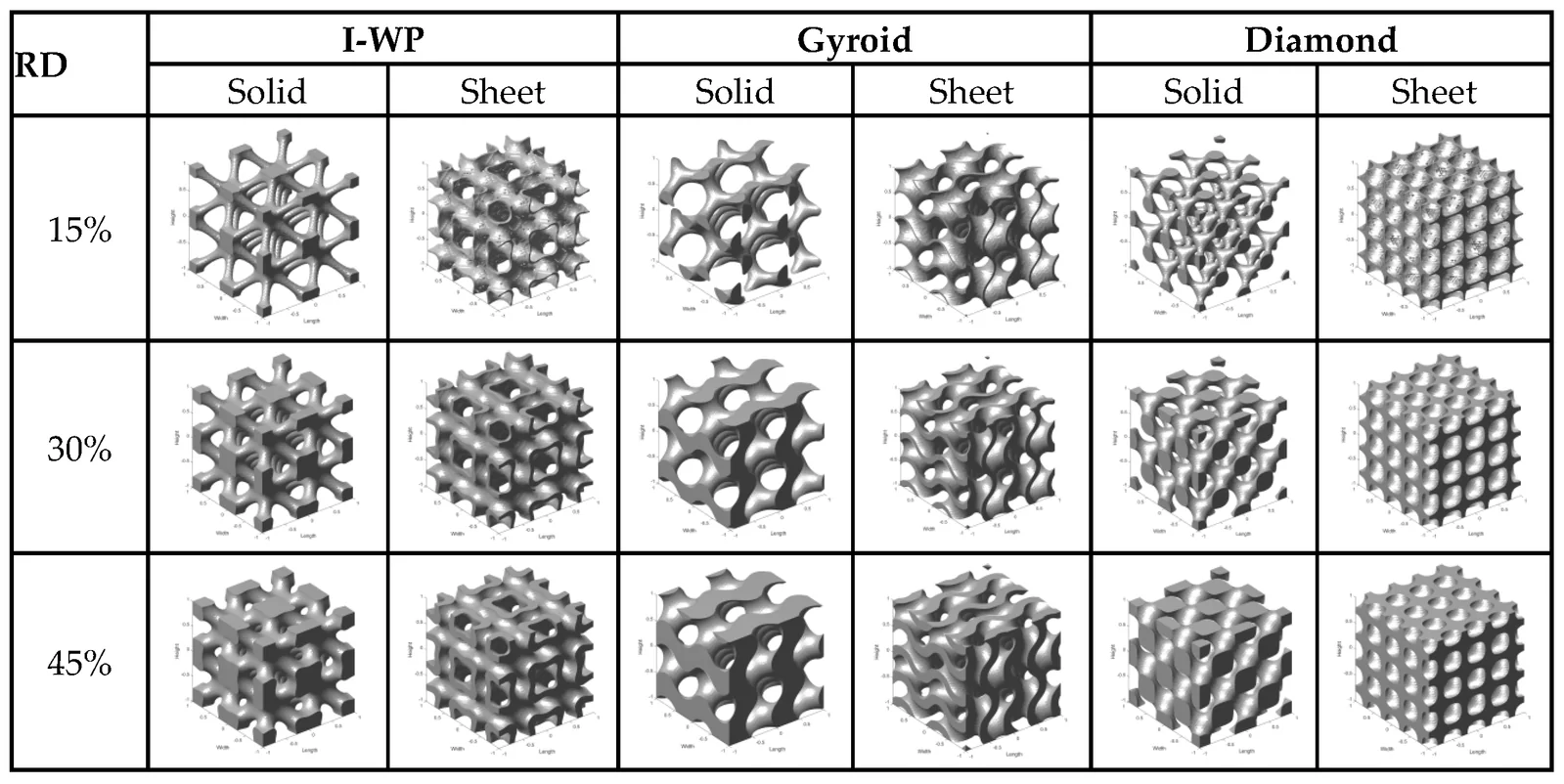
Skeleton (solid) vs. sheet TPMS structures at different relative densities.

The current advances in additive manufacturing have made it possible to fabricate complex porous structures, such as triply periodic minimal surfaces (TPMS). These structures represent one of the most promising classes of mechanical metamaterials due to their continuous topology, high controllability of geometric parameters, and the ability to tailor mechanical properties precisely. TPMS structures are a class of complex lattice geometries characterized by minimal surface area and zero mean curvature, which have attracted much attention in engineering and biological applications. These structures are particularly attractive for thermal management because of their excellent properties, such as a large surface area, fully interconnected fluid channels, and good mechanical properties, which collectively improve fluid flow and convective heat transfer performance [8,9]. Their smooth and continuous surfaces eliminate sharp corners and abrupt flow transitions, resulting in improved fluid flow distribution and reduced stress concentrations compared with conventional lattice structures. Several TPMS topologies have been investigated for thermal management applications, including gyroid, diamond, and I-WP structures [10,11]. The gyroid structure is characterized by a highly interconnected network and smooth flow passages that promote fluid mixing and heat transfer. Diamond structures exhibit a relatively uniform pore distribution and large surface area, often leading to favorable thermal performance. The I-WP geometry offers a distinct pore morphology and flow network that may affect both heat transfer enhancement and hydraulic resistance. Figure 1 illustrates several representative TPMS structures, such as the gyroid, I-graph wrapped package (I-WP) and diamond, based on solid and sheet networks [12,13]. These lattice geometries are generated with a relative density (RD) range of 15% to 45%. RD is a geometric parameter that indicates the fraction of solid material relative to the overall volume of the lattice. The higher the RD, the larger the solid fraction, which increases the available heat conduction pathways and expands the effective heat transfer surface area.

Baobaid et al. [14] conducted both numerical and experimental investigations to compare the performance of metal foam heat sink with that of a porous gyroid TPMS heat sink. Under identical conditions, the gyroid structure exhibited the higher Nusselt number despite an 18% increase in pressure drop. The findings indicated that the gyroid heat sink outperformed the metal foam in terms of cooling efficiency and provided more uniform temperature distribution. Saghir et al. [15] performed combined experimental and numerical analyses on thermo-hydraulic performance of metal foam and gyroid TPMS structures under different design parameters including heat fluxes and flow rates, with porosities of 0.7, 0.8 and 0.9. The metal foam Darcy–Brinkman model was combined with the energy equation for porous media, while Navier–Stokes’ formulation was combined with the energy equation for a gyroid structure. The results indicate that gyroid TPMS promotes a uniform temperature field by disrupting the thermal boundary layer. In contrast, metal foam exhibits non-uniform cooling with thermal boundary layer development along the flow. Although the gyroid exhibited 18% higher pressure drop, it demonstrated a superior Nusselt number and enhanced overall cooling performance compared to the metal foam. They also evaluated TPMS heat sinks with porosities of 0.7, 0.8, and 0.9 using printed porous aluminum. A porosity of 0.8 was identified as optimal for small surface cooling, offering balancing thermal performance and pressure drop. Buonomo et al. [16] carried out experimental work on the heat dissipation process in a porous medium under impinging jet flow conditions. Their results indicated that heat removal was enhanced in the presence of metal foam. However, the incorporation of metal foam also led to an increase in friction factor and a decline in the pressure gradient.

Further, Tang et al. [17] investigated the convective heat transfer performance of TPMS structures, including diamond, gyroid, and I-WP, in comparison with conventional fin-based heat dissipation. Air was used as the working fluid, and the base surface was heated at a constant temperature of 373.15 [K]. The results showed an enhanced Nusselt number for TPMS structures compared to fin-based heat sinks; in particular, the diamond structure exhibited the highest heat transfer performance. The numerical simulation results were validated through experimental work. Rathore et al. [18] studied flow properties and heat exchange in porous media using numerical simulation. TPMS lattices shaped as gyroid, diamond, I-WP and primitive are generated with identical porosity. The study aimed to investigate the effects of lattice geometry, tortuosity, effective porosity, and microporosity on permeability and inertial drag factors. It was observed that the Darcy number increased with porosity, whereas the inertial drag factor followed the opposite trends and decreased with increased porosity. The results revealed that the inertial drag coefficient and conductivity value were determined by flow regimes and lattice types. Attarzadeh et al. [19] developed computational design and analysis of heat and mass transfer in heat exchangers based on gyroid, diamond, and primitive TPMS structures. They examined the combined effects of wall thickness, inlet velocity, and TPMS type on thermal efficiency, heat transfer coefficient, and pressure drop. The authors found that the increasing wall thickness improved the thermal performance of the heat exchanger. They also reported that the performance of TPMS-based heat exchangers was highly sensitive to lattice geometry and inlet velocity.

Al-Ketan et al. [20] investigated forced convection heat transfer in heat sinks designed with diamond and gyroid geometry, considering solid and sheet networks, using numerical and experimental approaches. The results showed that G-sheet exhibited the highest convective heat transfer coefficient and the lowest thermal resistance, while the D-Solid exhibited the highest thermal efficiency. Moradmand and Sohankar [21] analyzed the thermo-hydraulic efficiency of heat exchangers with Schwarz-P and gyroid under various porosities using combined numerical and experimental approaches. The findings revealed that the gyroid structure provided higher heat transfer performance and lower pressure drop than the Schwarz-P type. An increase in the Reynolds number increased both the heat transfer coefficient and pressure drop. The study showed that the gyroid geometry exhibited superior thermo-hydraulic efficiency compared to traditional heat exchangers, offering enhanced energy efficiency and potential for compact designs.

Later, Sun et al. [22] examined how porosity influenced the thermo-hydraulic behavior of gyroid TPMS structures through numerical analysis. Their findings indicated that a lower porosity value resulted in higher surface area, which in turn enhanced thermal performance. In addition, Cheng et al. [23] studied the effect of porosity on various TPMS networks, using numerical work that showed that primitive topologies yielded the lowest pressure drop. The I-WP and gyroid topologies exhibited enhanced thermal performance compared with a simple cubic core. Moreover, increasing porosity reduced the heat transfer coefficient due to lower flow velocities within the TPMS; however, the Nusselt number increased owing to changes in the characteristic length scale. Chen and Li et al. [24] employed porous media for temperature profiles in compact heat exchangers, reducing computational time to one-tenth of the original approach. An effective porous media model for heat sinks should ensure equivalence in both thermal and flow performance. Hawken et al. [25] experimentally investigated the pressure drop profile of a Schwarz–Diamond heat exchanger, demonstrating that the pressure drop decreased with increasing porosity and hydraulic diameter of the structure.

Several studies have demonstrated that selected TPMS configurations may exhibit auxetic behavior. Auxetic materials, characterized by a negative Poisson’s ratio [26,27,28,29,30], are attractive for applications requiring high mechanical resistance, effective energy absorption, structural stability [31,32,33], and the capability to withstand dynamic loads [34]. Auxeticity in TPMS structures is not a universal feature; it depends on the type of minimal surface, porosity level, wall thickness, and the parameterization method. Numerical and experimental studies have indicated that structures based on surfaces such as gyroid, diamond, I-WP, or Neovius may exhibit a negative Poisson’s ratio within specific loading conditions and geometric configurations [35,36]. The auxetic mechanism arises from the curved, three-dimensional network of channels, which causes lateral expansion during deformation instead of the classical contraction. This mechanism differs from traditional auxetic metamaterials, such as re-entrant, chiral, or rotating-square structures, in which the effect arises from discrete element kinematics [37,38].

Unlike classical auxetic structures, TPMS offer continuous material networks with higher mechanical stability and additive manufacturability. Novak et al. proposed an image-modulated implicit-field method for designing auxetic TPMS structures. The developed functionally graded re-entrant TPMS exhibited stable auxetic behavior under large deformations, while porosity gradation enhanced deformation control, energy absorption, and impact mitigation performance [39,40]. Moreover, auxetic TPMS structures exhibit enhanced damage resistance, high energy absorption, and improved impact performance, making them suitable for lightweight and protective engineering applications.

The current study aimed to present the numerical evaluation of the thermo-hydraulic performance of various porous TPMS structures, considering the effects of relative density, structure morphology, inlet velocity and porosity. TPMS structures are characterized by zero mean curvature, where the sum of the principal curvatures at every point on the surface is equal to zero. These minimal surfaces can be periodically repeated with varying relative densities to obtain the desired architectural characteristics. Although previous studies have investigated TPMS topology, porosity, and flow conditions using solid-network representations, the coupled effects of TPMS topology, network morphology, relative density, and inlet velocity on thermo-hydraulic performance remain largely unexplored within a porous medium framework. The present study addresses this gap by systematically investigating these coupled parameters based on a porous matrix representation of the TPMS structure. Table 1 shows the implicit mathematical equations describing the unit cell of TPMS structures used in the present study. The details of these equations are described in the previous work by Iticha and Stręk [41] and Yeranee and Rao [12]. The variables α, β, and γ denote constants related to the unit cell size (L) in the x, y and z directions, respectively; c is the offset parameter. For a single TPMS unit cell, c = 0, as illustrated in Table 1.

The paper is organized into four main sections: Introduction, Models and Methods, Results and Discussion, and Conclusions. A list of comprehensive references is included. The Models Section describes the computational setup, including the geometrical model and mathematical governing equations with corresponding boundary conditions. The Methods Section presents the finite element method (FEM) employed in the numerical analysis. The goal of the investigation was to evaluate the influence of three porous TPMS heat sink geometries (diamond, gyroid and I-WP), network morphology (sheet and solid), structural representation (solid and porous) and relative density (RD = 15%, 30%, and 45%) on the cooling performance of heat sinks. This was examined through analysis of temperature and velocity fields, pressure drop, average Nusselt number, and Darcy friction factor. The results confirm that TPMS topology significantly influences thermal and hydraulic characteristics.

## 2. Materials and Methods

### 2.1. Numerical Model and Boundary Conditions

**Figure 2:**
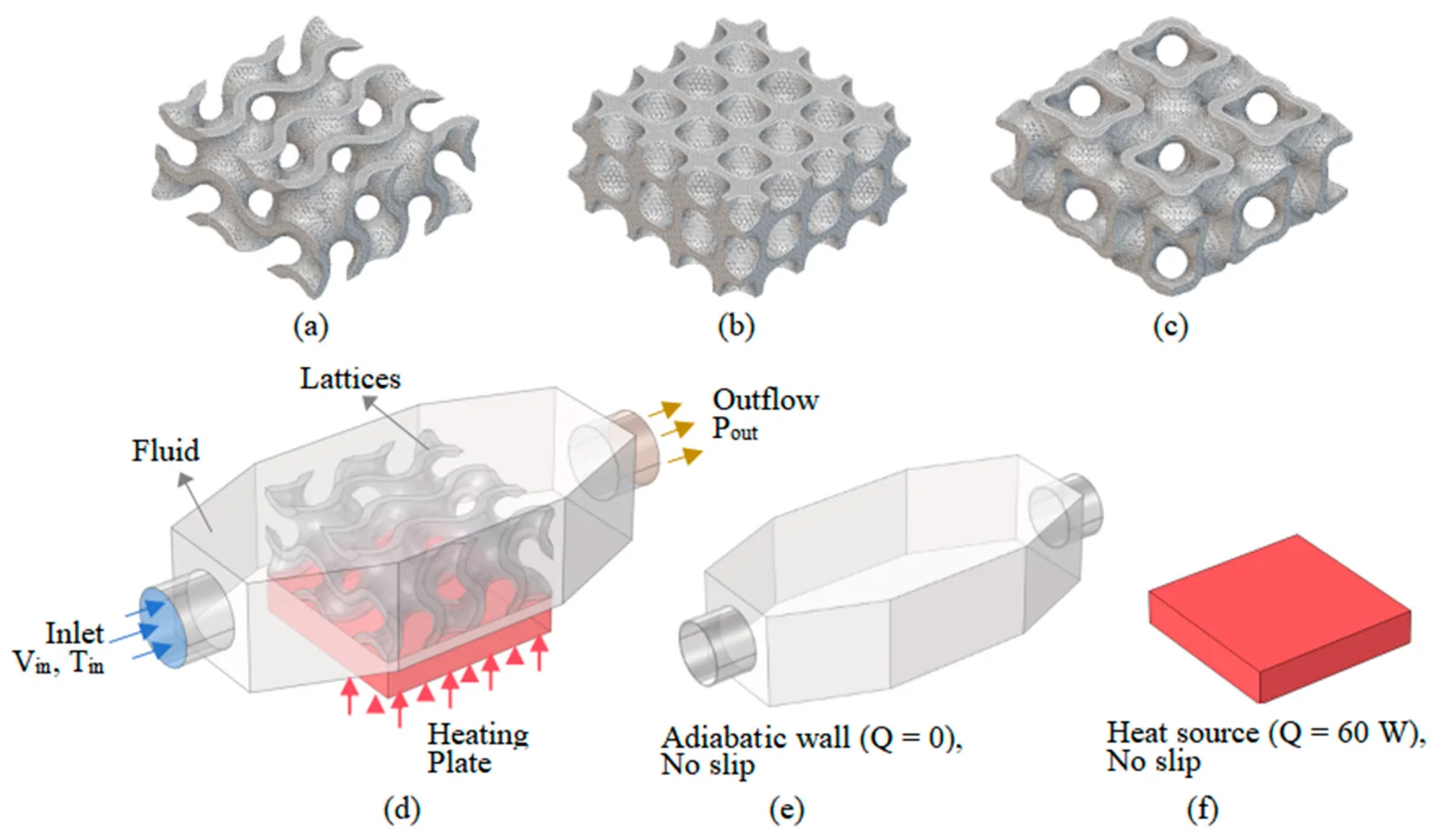
3D simulation model and boundary conditions: (**a**) gyroid, (**b**) diamond, (**c**) I-WP, (**d**) full model overview, (**e**) duct outer wall and (**f**) heat source.

The components of the complete heat sink model and the imposed boundary conditions are illustrated in Figure 2. Initially, three TPMS-based structures, each measuring 30 × 30 × 15 mm, were generated using MSLattice version 1.0 [20]. Extensions of 20 mm were added to both ends of each structure and filleted to facilitate fully developed fluid flow conditions and minimize pressure losses. Cylindrical sections with a diameter of 6 [mm] and a length of 8 [mm] were incorporated at both ends. The TPMS solid matrix was composed of aluminum, and a copper heating plate with dimensions of 30 × 30 × 6 [mm] was modeled at the bottom to provide a uniform heat flux. In addition, water was used as the working fluid for cooling the hot surface. In the current work, the material properties were assumed to be constant, and these properties are summarized in Table 2. The full 3D computational domains of HS were constructed in different software packages. The TPMS lattice domain was generated in MSLattice, while the heat source and fluid channel were modeled in COMSOL Multiphysics version 5.1 [15,42].

In the current numerical simulation, the working fluid (water) entered the channel with a constant inlet velocity (u_in_ = 0.05 [m/s]) and inlet temperature (T_in_ = 293.15 [K]), while the outlet boundary was treated as a pressure outlet (P_out_ = 0 [Pa]). In addition, a uniform heat flux corresponding to a constant input power of 60 Watts was applied at the bottom surface of the solid domain, whereas all remaining surfaces were assumed to be adiabatic, with no-slip wall conditions.

The boundary condition can be expressed as follows:

- For the inlet channel boundary:
  (1) u = u_in_, v = 0, w = 0,T = T_in_,
- For the outlet channel boundary:
  (2) P = P_out_,
- For fluid–solid interfaces:
  (3a) u = 0, v = 0, w = 0,
  (3b) T_s_ = T,
- For the bottom heat sink surface:
  (4) $$q_{w}=\frac{\partial \mathrm{Ts}}{\partial z}=\mathrm{const}.$$

In the equations above, u, v, and w denote the velocity components along the x-, y-, and z-axes, respectively. The terms u_in_ and T_in_ represent the inlet velocity and ambient temperature, while P_out_ corresponds to the outlet pressure of the coolant. The parameter q_w_ indicates the heat flux applied at the bottom wall.

### 2.2. Governing Equations

The CFD simulation model employed in this study is illustrated in the above Figure 2d. The bottom surface of the model was subjected to a uniform, constant heat source, while the working fluid entered through the inlet side and exited through the outlet section on the opposite side. The numerical simulations were performed using COMSOL Multiphysics v. 5.1 software [42] to resolve heat transfer and fluid flow in the heat sink model.

During analysis, certain assumptions were made: (1) the fluid flow was steady and incompressible, (2) the porous medium was homogeneous and had uniform porosity, (3) there was local thermal equilibrium between the fluid and the solid phase of metal foam, and (4) no heat generation was considered within the porous medium.

Geometrical porosity, ε_g_, is an intrinsic morphological property of the generated TPMS structure and is defined by its solid-to-total volume ratio. Accordingly, it is directly related to the relative density as (ε_g_ = 1 − RD). In contrast, the porosity employed in the homogenized porous medium formulation, (ε), represents a model parameter introduced in volume-averaged governing equations to characterize the effective fluid volume fraction within the equivalent porous domain.

In the present numerical model, the value of porosity (ε = 0.8) was prescribed for the porous medium formulation and maintained constant across the investigated cases. Therefore, (ε) is distinguished from the geometrical porosity determined by the relative density of the explicitly generated TPMS structure. The effective porosity of the porous TPMS structure can be computed using the formula ε_eff_ = (1 − RD)∙ε.

The fluid flow within the open channel region was described using the Navier–Stokes equation (Equation (5)), whereas the Brinkman equation (Equation (6)) [43,44] was employed to determine the flow through the channel filled with metal foam. In addition, the continuity equation is given by Equation (7), while the energy equations describing heat transfer in the solid domain, aluminum foam region, and fluid domain are represented by Equations (8)–(10), respectively:

(5) 0 = ∇·(−p**I** + μ(∇**u** + (∇**u**)^T^) + **F**),

(6) $$\frac{\rho }{\varepsilon }\left(\left(u\cdot \nabla \right)\frac{u}{\varepsilon }\right)=\nabla \cdot \left(-pI+\frac{\mu }{\varepsilon }\left(\nabla u+({\nabla u)}^{T}\right)-\left(\frac{\mu }{K}+\beta_{f}\left|u\right|\right)u+F\right),$$

(7) ∇·(*ρ***u**) = 0,

(8) ∇·(−k_s_∇T)= 0,

(9) ρc_p_**u**·∇T= ∇·(k_eff_∇T),

(10) ρc_p_**u**·∇T= ∇·(k_f_∇T),

where p, T, and **u** stand for the pressure, temperature, and velocity vector of the flow, respectively; c_p_, ρ, k_f_ and k_s_ are the specific heat capacity of the fluid, density of water, thermal conductivity of water, and thermal conductivity of water, respectively. β_f_ represents the Forchheimer coefficient, ε is the porosity, and K represents the permeability of aluminum.

The effective thermal conductivity (k_eff_) of the aluminum foam filled with water was determined using the porosity (ε) and thermal conductivity of the solid phase (k_s_) and fluid phase (k_f_) by applying the rule of mixtures as described in the previous paper by Iticha and Strek [41] and Calmidi et. al. [45]:

(11) k_eff_ = ε · k_s_ + (1 − ε) · k_f_.

A set of evaluation metrics was defined to evaluate fluid flow, convective heat transfer characteristics and the overall thermal performance of the TPMS heat sink. The convective heat transfer coefficient (h) was used for quantifying the effectiveness of heat transfer between the solid wall and fluid, as defined in Equation (12) [46]:

(12) $$h=\frac{q^{\prime \prime }}{T_{m}-T_{\mathrm{in}}},$$

where q″ represents the applied uniform heat flux of 6.67 [W/cm^2^], which was calculated from the generated thermal power of 60 [Watts] distributed over a heating surface area of 30 × 30 [mm]. T_m_ and T_in_ denote the local surface and fluid temperatures, respectively. T_m_ was measured on the heating plate 1 mm below the interface.

Equation (13) describes the local Nusselt number, a dimensionless parameter that characterizes the effectiveness of convective heat transfer relative to conductive heat transfer within a fluid:

(13) $$\mathrm{Nu}=\frac{hD_{h}}{k},$$

where k is the thermal conductivity of water, and D_h_ is a characteristic length, which was determined as four times the ratio of void volume (v_void_) to the wetted surface area of the structure (A_s_), as shown in Equation (14) [47]:

(14) $$D_{h}=\frac{4v_{\mathrm{void}}}{A_{s}}$$

In heat sink performance analysis, the friction factor is another dimensionless parameter used to evaluate the flow resistance and pressure drop caused by fluid flow through the channels, and it is defined by Equation (15) [48,49]. Moreover, the overall thermo-hydraulic performance index of the TPMS structure (α) was determined by combining the dimensionless parameters f and Nu, as expressed in Equation (16), where Δp, L, ρ, and u represent the pressure loss, length of TPMS lattice, fluid density, and flow velocity, respectively:

(15) $$f=\frac{0.5\Delta pD_{h}}{{L\rho u}^{2}},$$

(16) $$\alpha =\frac{\mathrm{Local Nu}}{\sqrt[{3}]{f}}.$$

The equations above fall into two categories. Equations (5)–(10) are governing conservation equations expressing fundamental mass, momentum, and energy balances. Equations (12)–(16) are definitional relations that quantify heat transfer and flow performance, such as the convective heat transfer coefficient, Nusselt number, hydraulic diameter, friction factor, and thermo-hydraulic performance index, directly from the computed field variables. Equation (11), the effective thermal conductivity, is an empirical rule-of-mixtures correlation adopted from Iticha and Strek [41] and Calmidi et al. [45]. All governing, definitional, and empirical relations employed in this study were derived from the established literature, as indicated by the corresponding citations, and none are original contributions of the present work. The novelty of this study instead lies in the porous medium modeling framework, in which the coupled effects of topology, relative density, and structural representation on thermo-hydraulic performance were systematically evaluated.

### 2.3. Mesh Independence Analysis

**Figure 3:**
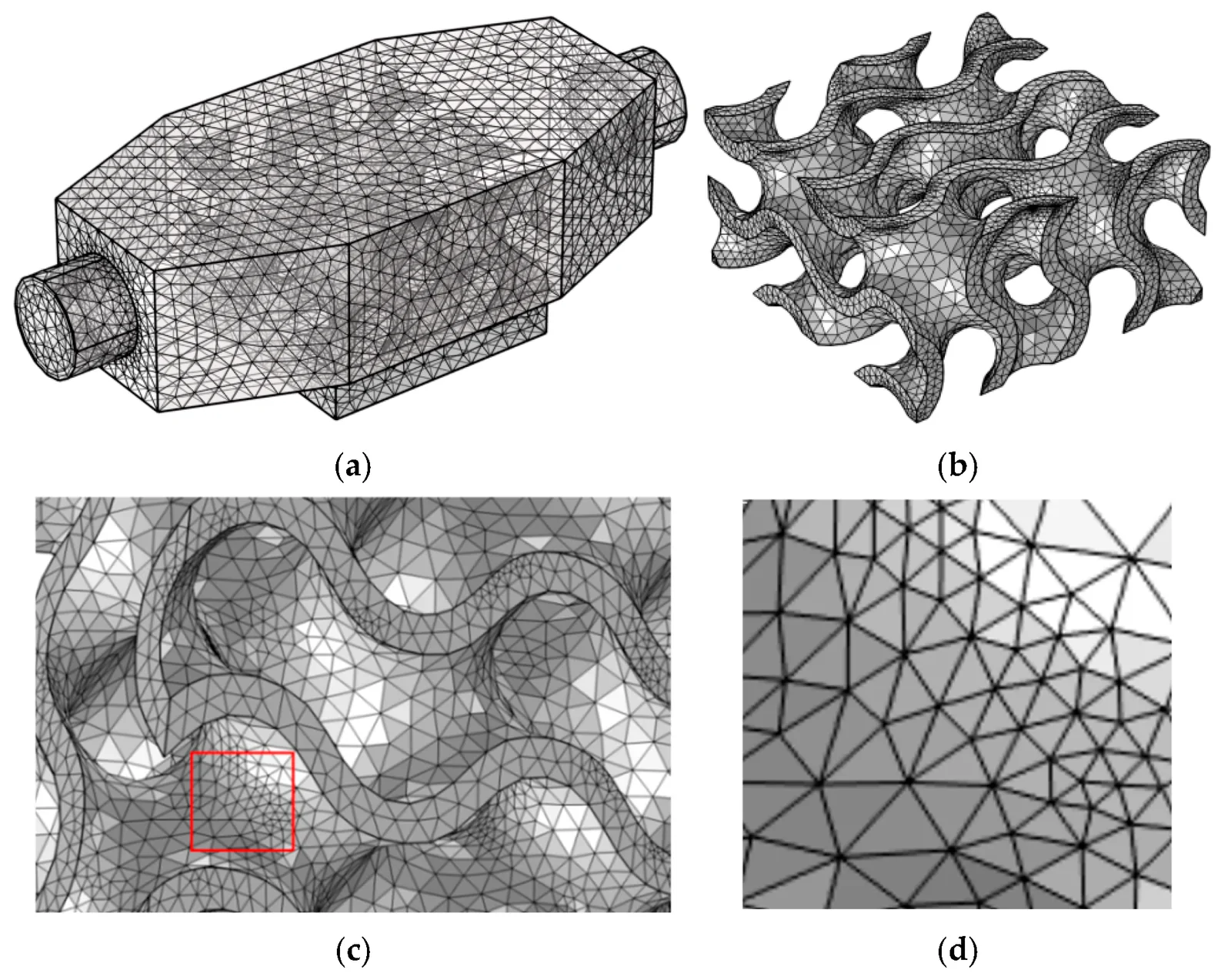
(**a**) 3D full model mesh generation; (**b**) meshes in gyroid structure; (**c**) local view of the meshes; (**d**) enlarged view of the mesh.

The CFD model of the heat sink structure was meshed in COMSOL Multiphysics version 5.1 software using unstructured tetrahedral elements for both the solid and fluid domains, while hexahedral elements were employed for the copper plate (see Figure 3). Polyhedral mesh elements were used, incorporating five boundary-layer elements with a growth factor of 1.2 at the solid–fluid interface. A mesh convergence study was performed to ensure accurate and reliable numerical results.

**Figure 4:**
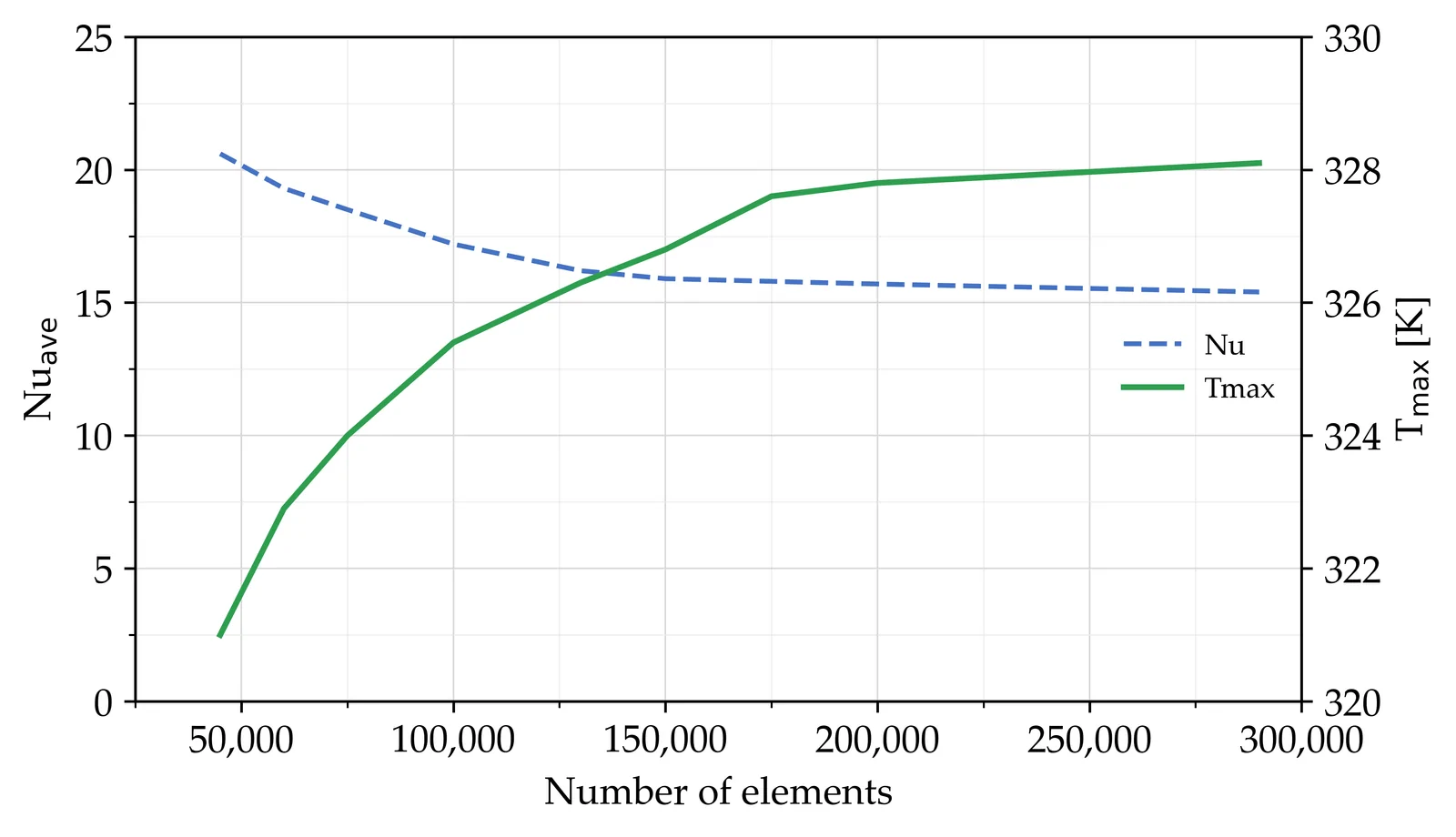
Mesh sensitivity analysis results.

Several numerical simulations were conducted using different mesh sizes, with the average Nusselt number and maximum temperature considered as the evaluation criteria. Figure 4 illustrates that between the two finest meshes evaluated (166,511 and 293,667 elements), the relative deviation in the average Nusselt number was approximately 3.3%, which was considered acceptable for confirming mesh independence. The mesh with 293,667 elements was therefore adopted for all subsequent simulations. This approach follows the relative error convergence formulation of Hajialibabaei et al. [50] (Equation (17)), in which the terms u, v, and w represent the fluid velocity components in the x, y, and z directions, respectively.

(17) $$R_{c}=\frac{1}{n\cdot m}\sum_{i=1}^{i=m}\sum_{j=1}^{j=n}\left|\frac{F_{i,j}^{s+1}-F_{i,j}^{s}}{F_{i,j}^{s+1}}\right|,$$

where (i,j) represents the grid’s coordinates indexes, F and s denote the independent parameters, and the number of iterations, respectively.

## 3. Results

### 3.1. Surface Temperature Distribution

**Figure 5:**
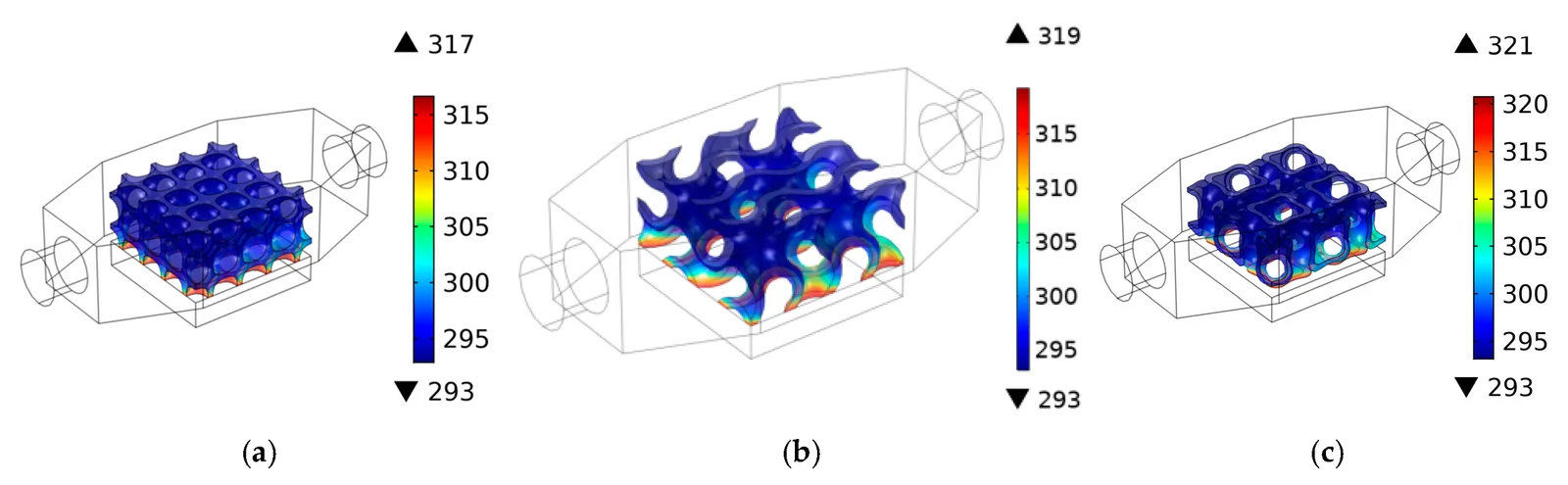
Surface temperature field for various geometries: (**a**) diamond, (**b**) gyroid and (**c**) I-WP.

Figure 5 presents the temperature field within diamond, I-WP, and gyroid porous TPMS heat sinks. I-WP heat sink shows the highest localized temperature reading 321 [K] near the heat source and the lowest temperature at the inlet surface of the channel. This indicates the limited temperature distribution and inefficiency in removing heat from the sources. In contrast, the temperature contour of the diamond TPMS geometry maintains the lowest value, with the maximum value of 317 [K] also near the heat source, demonstrating superior thermal performance. The gyroid heat sink demonstrates an intermediate thermal gradient with the peak value of 319 [K], despite it spreading more uniformly throughout the structure. This indicates enhanced heat dissipation efficiency and improved fluid circulation within the porous medium.

**Figure 6:**
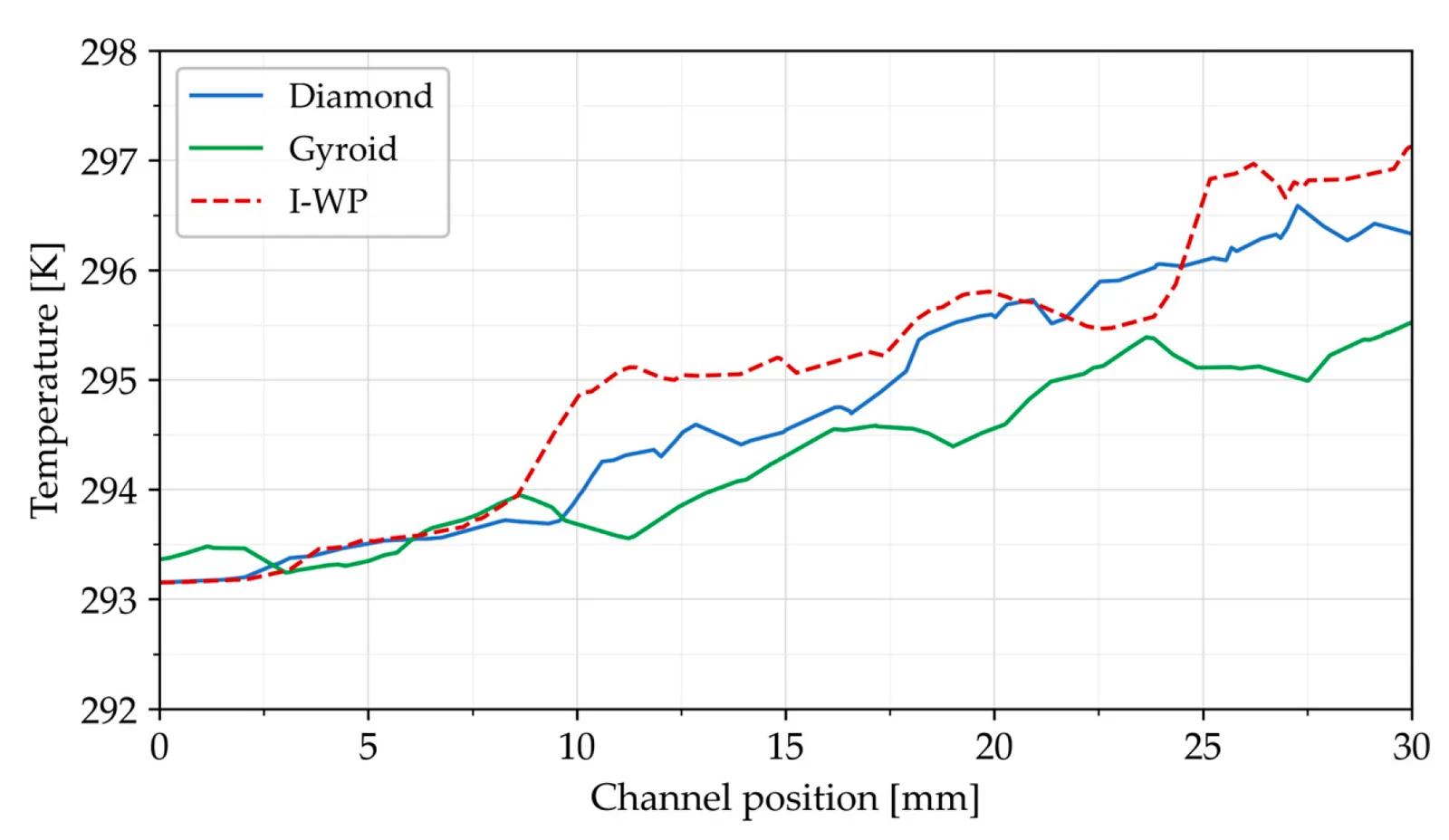
Temperature distribution along the channel length.

Figure 6 shows the temperature distribution along a line passing through the center of the TPMS structure for all three designs. In all models, the sinusoidal rising shape of the plot indicates variations in the temperature profile within the lattice structures. Lower temperatures are observed in the free-channel region. For all lattice structures, the temperature increased from 293.15 [K] at the inlet to higher values at the outlet due to continuous heat absorption by the coolant as it passed through the heated porous domain. Among the tested structures, the gyroid lattice exhibited the consistently lowest temperature distribution throughout the channel length. The outer surface temperature of the gyroid structure reached approximately 295.5 [K], which is lower than that observed for the diamond and I-WP structures. These results indicate that the gyroid geometry dissipated heat more efficiently and improved thermal management.

### 3.2. Fluid Flow Characteristics

**Figure 7:**
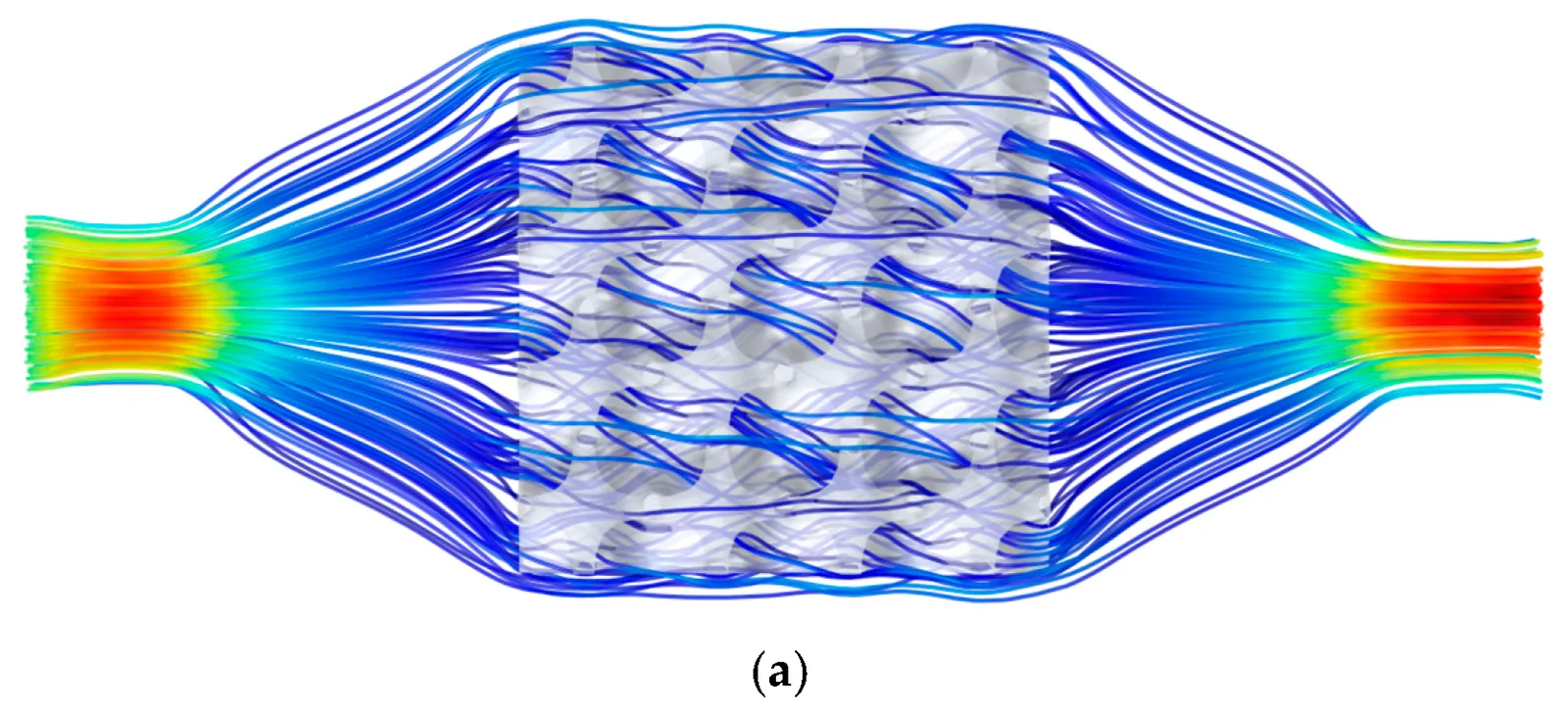

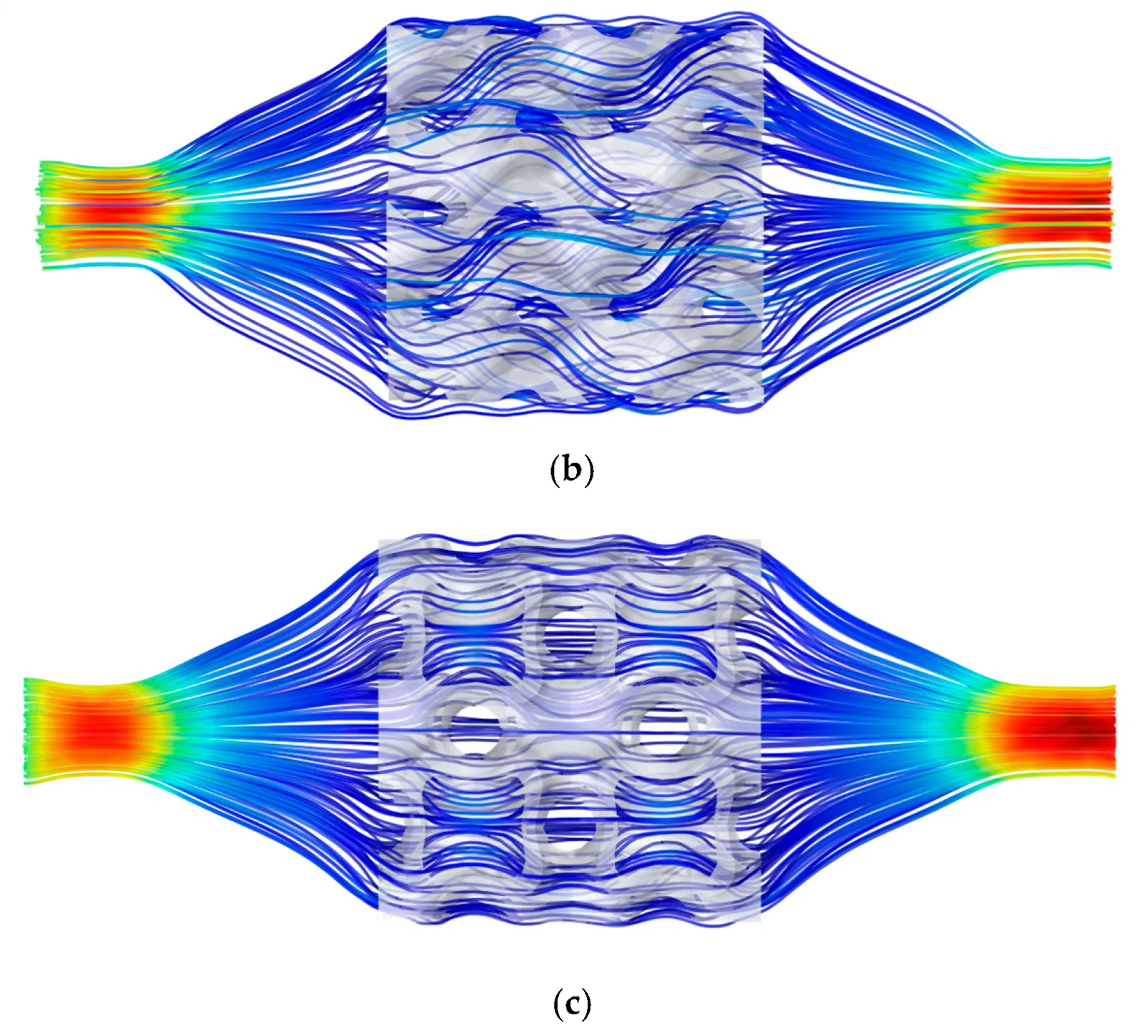
Fluid flow pattern within the TPMS lattice: (**a**) diamond (**b**) gyroid and (**c**) I-WP.

Figure 7 illustrates the velocity streamlines obtained from the 3D numerical simulations of the TPMS lattices. Among the heat sink models, the gyroid structure featured a twisted, turning, and interconnected passage that extended throughout the porous domain. However, the continuous channel geometry of the gyroid increased flow mixing and enhanced convective heat transfer coefficient. The diamond configuration demonstrated the most uniform and least obstructed flow characteristics, indicating superior permeability and reduced flow resistance. It demonstrated a higher max velocity of 0.1003 [m/s]. Meanwhile, the I-WP topology generated stronger flow disturbances and localized stagnant regions, resulting in less uniform hydrodynamic behavior.

For all investigated models, the channel cross-sectional area varied along the flow direction, with reduced cross-sectional areas near the inlet and outlet regions and expanded at the central region. The fluid velocity increased in the region of lower flow to satisfy the conservation of mass (continuity principle). This changed in cross-section-induced disturbances in velocity magnitude and direction of fluid flow. Therefore, higher velocity was observed at the inlet and outlet sections of the heat sink channels, as depicted in Figure 7.

**Figure 8:**
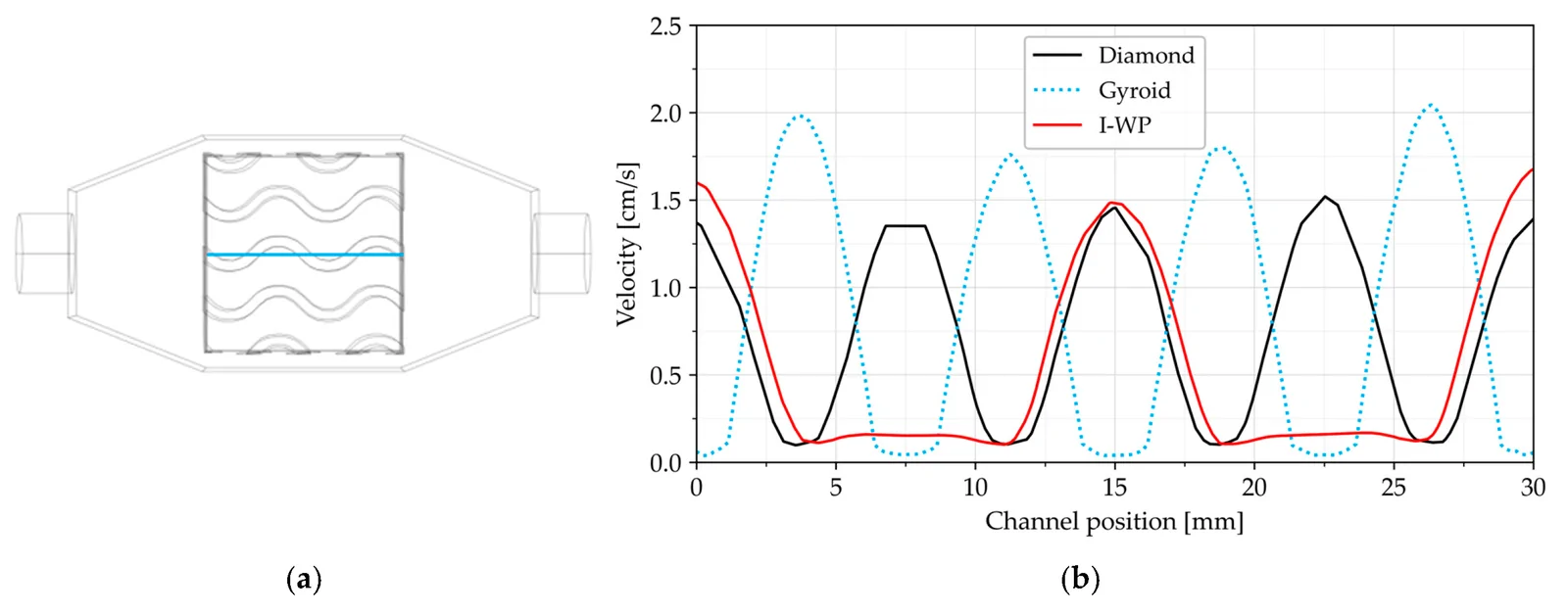
(**a**) Schematic of centerlines within the TPMS domain and (**b**) velocity profile along the channel.

The plot in Figure 8 shows the velocity field along the centerlines of the TPMS structure domain. In all models, the periodic oscillation velocity profiles correspond to the repeating unit-cell structural geometry. Among the investigated geometries, the gyroid structure demonstrated the most oscillatory velocity profile, with the peaks reaching approximately 2 [cm/s]. This indicates that the presence of tortuous flow passages repeatedly contract and expand the fluid stream. This behavior promotes the strongest flow mixing and highest local velocities, which enhance convective heat transfer at the expense of increased hydraulic losses.

### 3.3. Pressure Gradient

**Figure 9:**
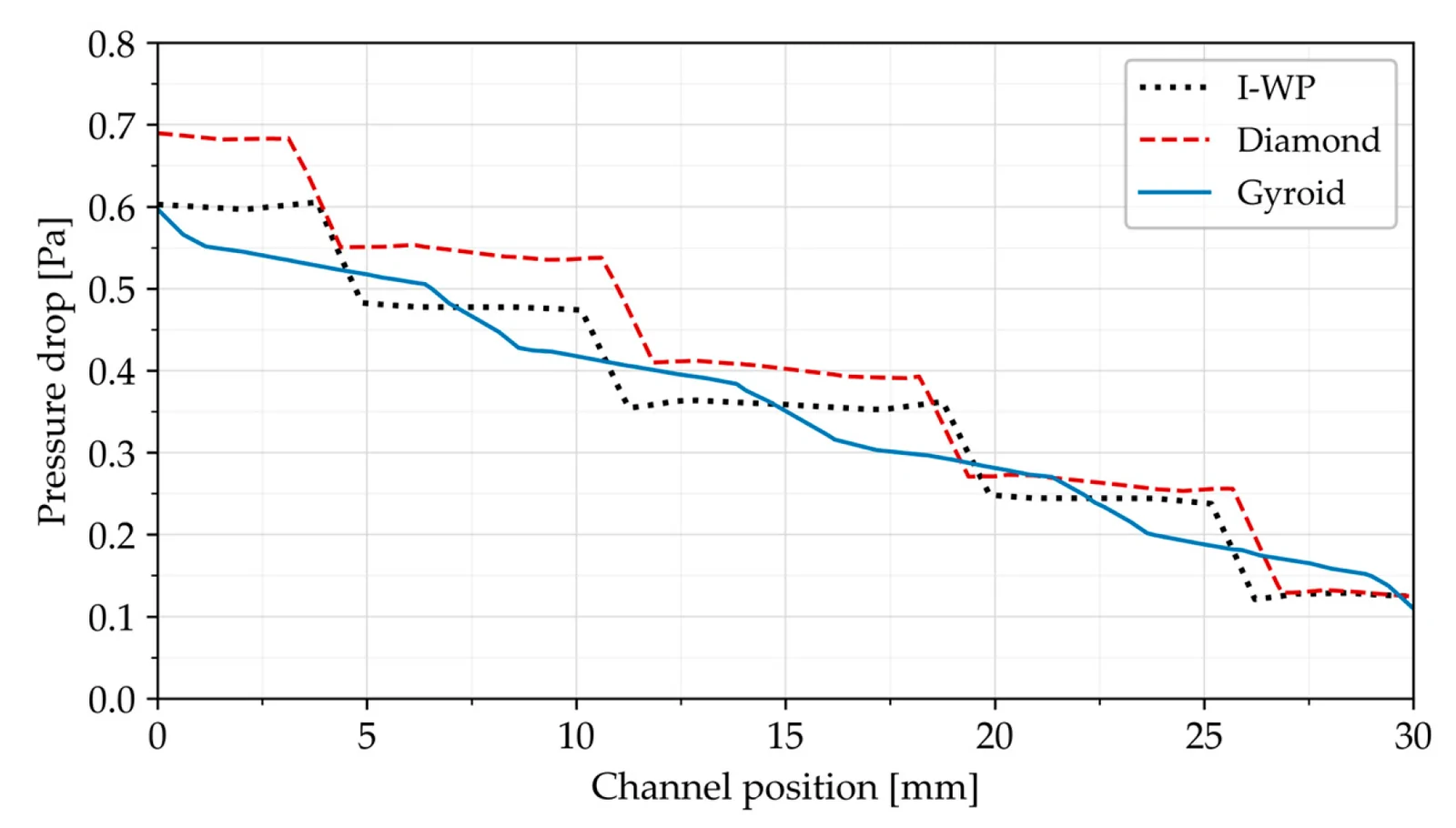
Pressure gradient along the centerline of: gyroid, diamond, and I-WP.

Figure 9 presents the pressure drop profiles along the centerline of the porous TPMS structure, illustrating the variation in average pressure. The results were obtained from numerical simulations conducted at an inlet velocity of 5 [cm/s] and a heat source of 60 [W]. In all models, the pressure monotonically decreased from the inlet to the outlet surface of the structure due to flow resistance within the porous matrix. Among the configurations, the diamond lattice exhibited the largest pressure drop, averaging 0.72 [Pa], indicating the highest hydraulic resistance. The sharp pressure drop reflects significant hydraulic resistance to fluid flow, potentially resulting in higher pumping power requirements and reduced heat dissipation efficiency. Conversely, a lower pressure gradient suggests diminished flow resistance, facilitating more uniform pressure variation and enhanced thermo-hydraulic performance. This is due to the surface configuration of the diamond structure creating greater flow resistance compared to the gyroid and I-WP. It shows that interconnected pores of diamond arrangements impose significant fluid resistance.

The gyroid HS reached the highest maximum pressure of about 0.58 [Pa], but it demonstrated more evenly distributed pressure along its structure. This structure exhibited the lowest and smoothest pressure drop profile among the configurations. The continuous surface morphology enables smoother fluid transport and more efficient pressure recovery between successive pore sections. The superior performance of the gyroid topology can be attributed to its smooth and continuous flow pathways, which reduce frictional resistance and result in a more uniform pressure gradient. The pressure drop across the I-WP was more moderate compared to the gyroid and diamond, with a maximum of 0.602 [Pa].

### 3.4. Local Nusselt Number

**Figure 10:**
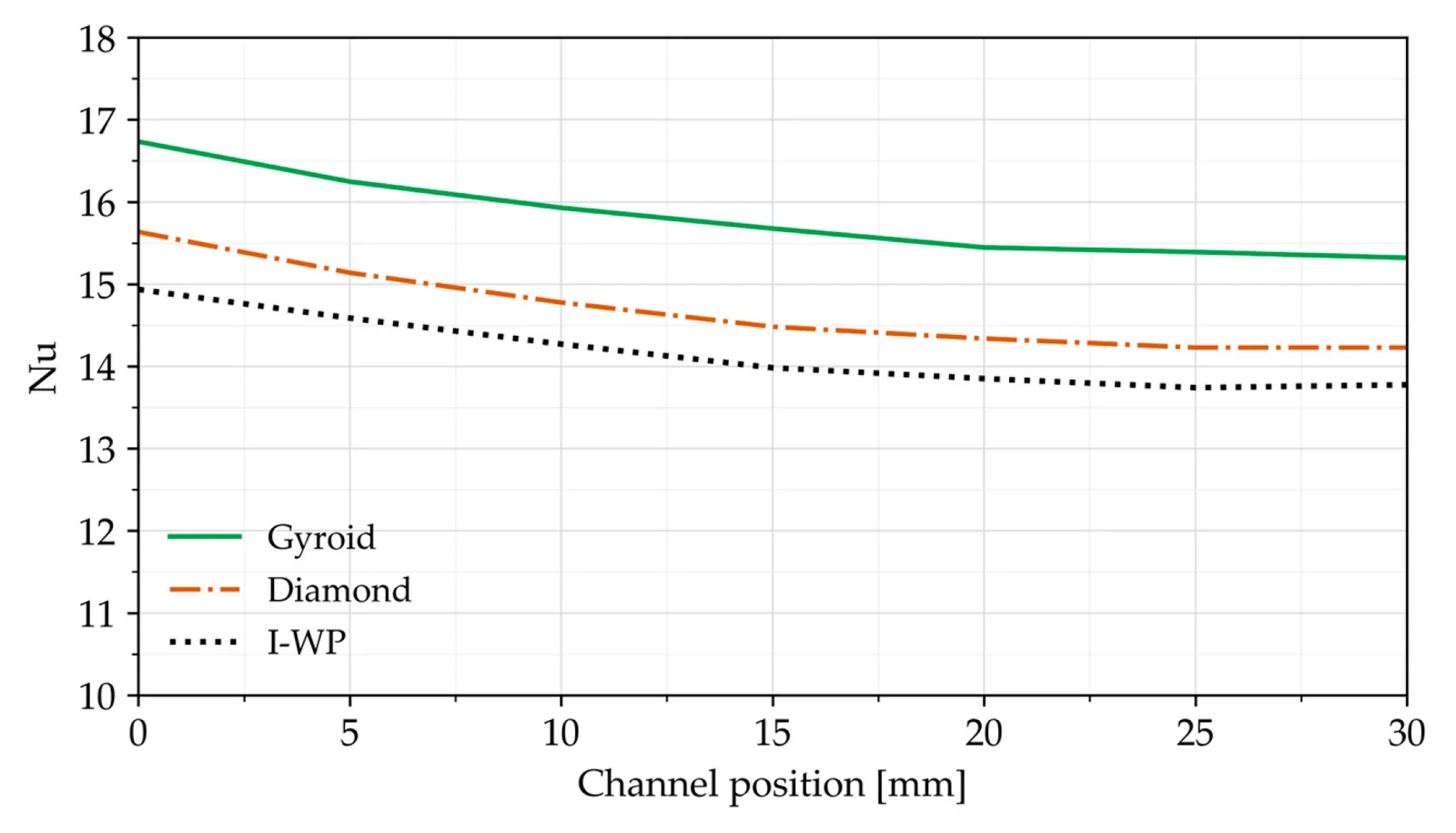
Variation in Nusselt number along the flow direction.

Figure 10 presents the variation of the local Nusselt number along the flow direction for different heat sink geometries under the conditions of an inlet velocity of 5 [cm/s] and a porosity level of 0.8. During the Nu calculation, the local temperature (Tb) was measured at a location 1 mm below the interface between the heat source and the TPMS structure. As illustrated in Figure 10, the Nu decreased gradually for all geometries due to thermal boundary layer development and reduction in the fluid–solid temperature gradient, which weakened convective heat transfer downstream. Furthermore, compared with the I-WP geometry, the gyroid and diamond models exhibited average Nusselt number enhancements of 10.9–12.4% and 2.9–4.7%, respectively. The gyroid structure exhibited the largest Nusselt number, ranging between 15 and 17 across the lattice length, which further highlights its enhanced convective heat transfer in comparison with other structures. The Diamond model also has good thermal behavior, although its performance was a few percent lower than that of the gyroid geometry. These results reveal that the gyroid TPMS offers the most effective heat dissipation and promotes a more uniform cooling distribution within the porous matrix.

### 3.5. Fluid Flow and Temperature Contour in Solid and Porous Gyroid Structure

**Figure 11:**
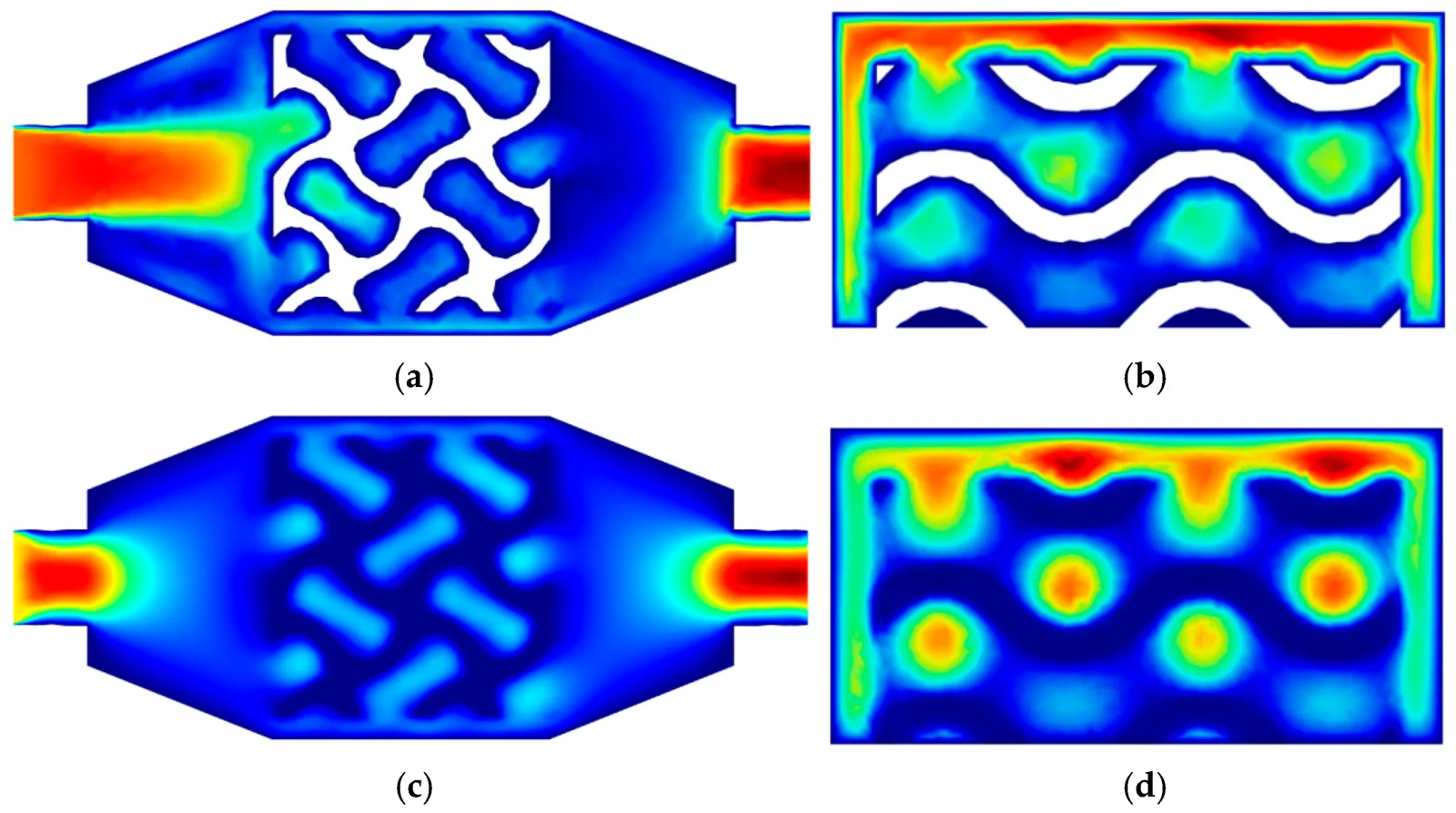
Velocity contour distributions at the mid-plane section: (**a**) solid (XY plane), (**b**) solid (YZ plane), (**c**) porous (XY plane), and (**d**) porous (YZ plane).

To compare the velocity distribution at the selected sections (Figure 11), the flow fields at the XY and YZ mid-planes were analyzed for both porous and solid gyroid structures. During the simulations, heat transfer and fluid flow in the solid TPMS were determined by the energy equation coupled with the Navier–Stokes equations. However, the heat transfer and fluid transport within the porous TPMS matrix were described using the energy equation and the Brinkman equations, respectively. To ensure a fair comparison, the dimensions of domains and all other input parameters were kept identical for both the solid and porous configurations. In the porous matrix model, the porosity and permeability values were specified as 0.8 and 1 × 10^−8^ [m^2^], respectively. The velocity contour visualization shown in Figure 11 enables a clear understanding of the flow patterns within the structures. In the XZ cutting plane (Figure 11a,c), the fluid enters the channel with a high velocity (red region) and subsequently splits around the solid ligaments. Localized low-velocity regions developed due to flow separation and re-circulation within the enlarged flow passages. The solid gyroid walls obstructed the fluid flow path. In contrast, the porous gyroid structure facilitated fluid penetration through the interconnected pore network, leading to a more uniform velocity distribution. In the YZ plane (Figure 11b,d), the vertical flow behavior of both solid and porous gyroid structures shows higher velocities near the upper surface within the narrow passages, consistent with the continuity equation. The blue regions within the structures represent low velocity zones caused by flow separation in the expanded flow passages.

**Figure 12:**
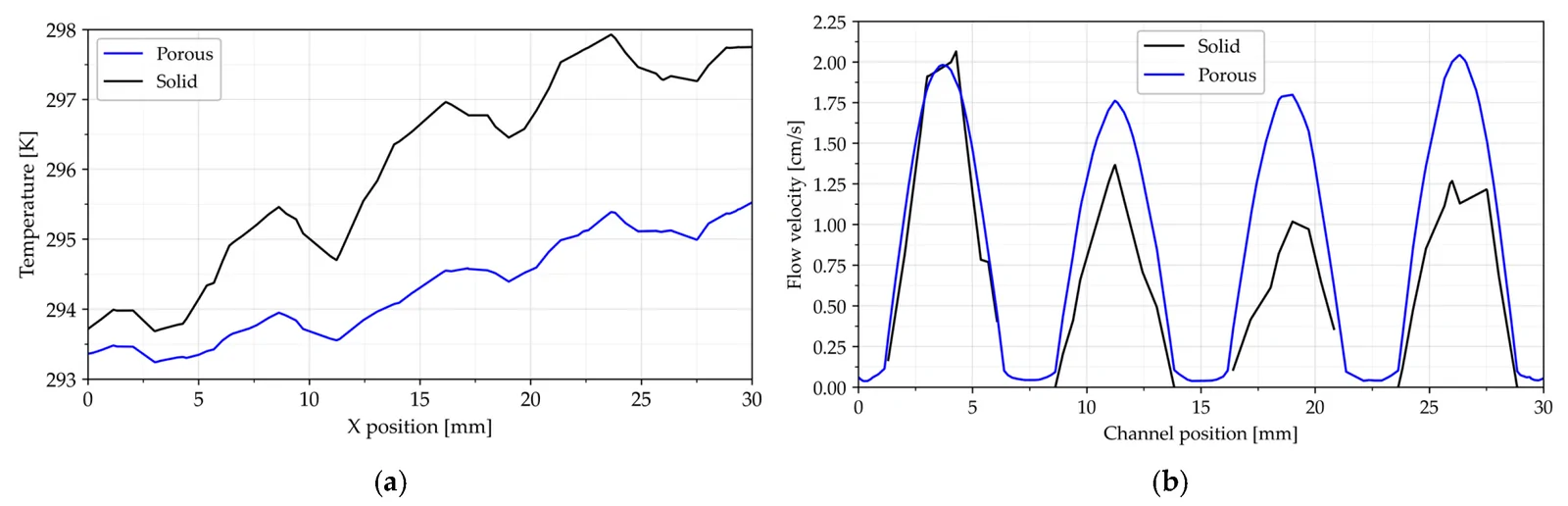
(**a**) Temperature field vs. channel position; (**b**) velocity profile vs. channel position.

Figure 11 compares the temperature and velocity contours along the longitudinal direction for the porous and solid gyroid TPMS heat sinks. In Figure 12a, the temperature results increase progressively along the flow direction for both heat sinks, reflecting the continuous absorption of thermal energy from the heat source. However, a clear distinction is observed between the two structures. The porous gyroid consistently maintained lower temperature throughout the entire domain compared with the solid type. This behavior is attributed to the highly interconnected pore network, which enhances convective heat transfer by increasing the solid–fluid interfacial area for thermal exchange. At the outlet of the structure, the solid heat sink reached a maximum temperature of 297.7 [K], whereas the porous type exhibited a peak temperature of 295.5 [K], representing a temperature reduction of 2.2 [K]. Figure 12b demonstrates the velocity plot along the flow direction for the porous and solid gyroid models. The periodic velocity fluctuations arise from the repeating lattice geometry, where flow acceleration occurs in open channels and deceleration occurs within constricted regions. The discontinuities in the black curve correspond to the solid wall of the lattice where fluid flow is absent. Both structures attain similar maximum velocities near the inlet (around 0.02 [m/s]). However, the porous gyroid maintains higher velocities in the downstream sections, with peak values ranging from 1.7 to 2 [cm/s]. In contrast, the solid gyroid exhibits a progressive decline in velocity, with peak values decreasing to approximately 1.4 [cm/s]. The interconnected pore network of the porous gyroid promotes improved fluid penetration and more uniform coolant distribution, thereby enhancing fluid transport throughout the structure. These results demonstrate that the porous gyroid architecture provides superior hydrodynamic performance, contributing to the enhanced thermal behavior.

### 3.6. Thermal and Fluid Flow Behavior in Sheet and Solid Gyroid Networks

**Figure 13:**
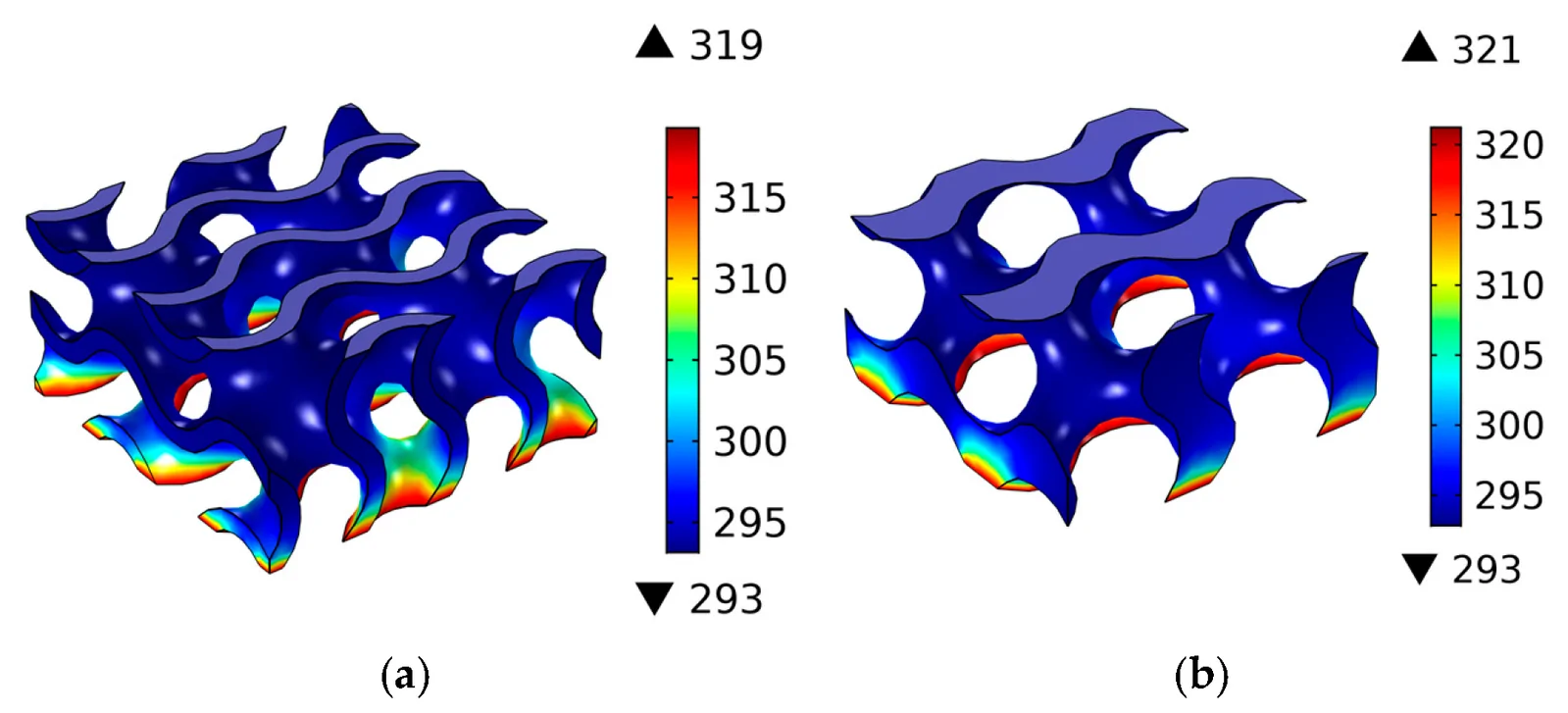
Temperature profile within gyroid structure: (**a**) sheet and (**b**) skeleton.

The 3D CFD simulation results presented in Figure 13 compare the thermal performance of the solid gyroid and sheet gyroid TPMS HS structures. The temperature contours reveal that the maximum value for the solid gyroid configuration was approximately 2 [K] higher than that of the sheet structure. This indicates that sheet gyroid (Figure 13a) heat sink achieved a lower peak surface temperature, corresponding to a reduction of approximately 0.58% compared with the solid configuration (Figure 13b). The improved thermal performance is attributed to the higher surface-area-to-volume ratio and enhanced fluid accessibility of the sheet-based TPMS geometry, which collectively increase the effective heat transfer area available for fluid–solid interactions and promote more efficient convective heat transfer. Consequently, convective heat transfer is enhanced, resulting in more uniform temperature distribution, improved heat dissipation efficiency, and reduced hot spot formation throughout the structure.

**Figure 14:**
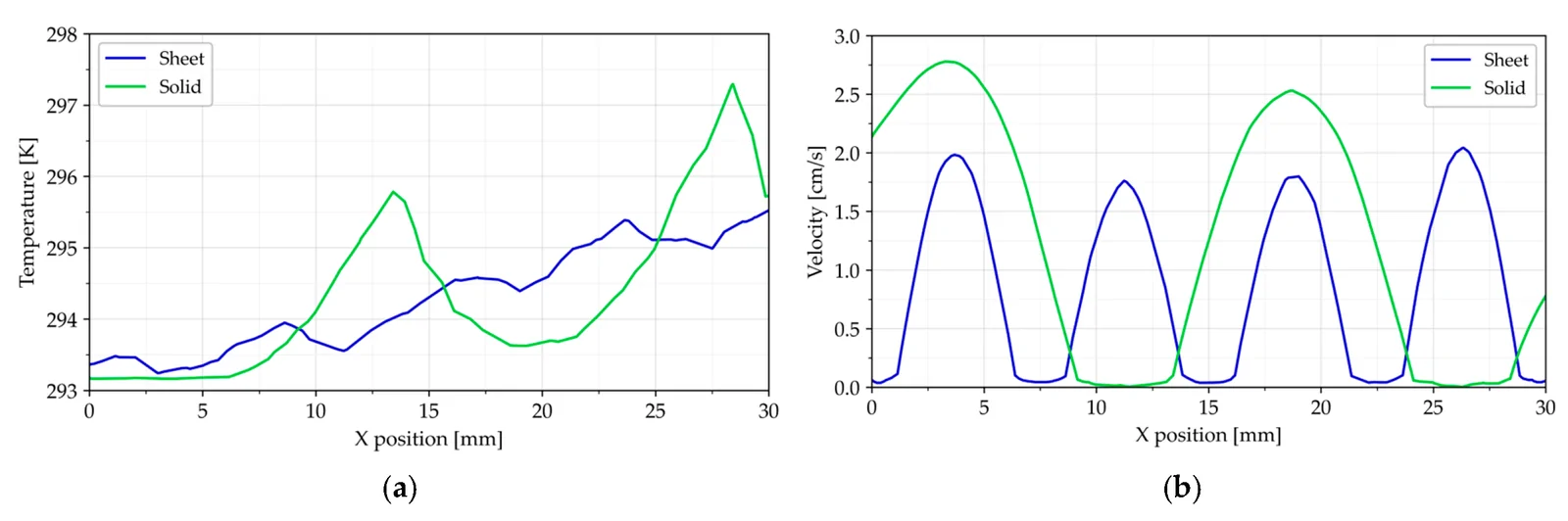
Temperature and velocity profiles as func (**a**) Temperature distribution versus channel position and (**b**) velocity field versus channel position.

The plot shown in Figure 14a indicates that the temperature profile along the center of the heat sink domain. The temperature field in the sheet gyroid was relatively uniform throughout the internal surfaces. The relatively larger surface area and complex geometry of the gyroid sheet tend to cause partial blockage within the flow passages. Figure 14b presents the velocity variation along the flow direction for the sheet and solid gyroid configurations. Both structures demonstrated periodic velocity fluctuations, reflecting the repeating nature of the gyroid unit cell. The region of high velocity corresponds to flow acceleration through narrow passages. The solid gyroid structure produced higher peak velocities, reaching 2.8 [cm/s]. However, the sheet gyroid exhibited 29% lower peak velocity than the solid gyroid, indicating a more distributed flow field that supports improved thermal uniformity.

### 3.7. Effects of Relative Density Variation on Thermal and Fluid Flow

**Figure 15:**
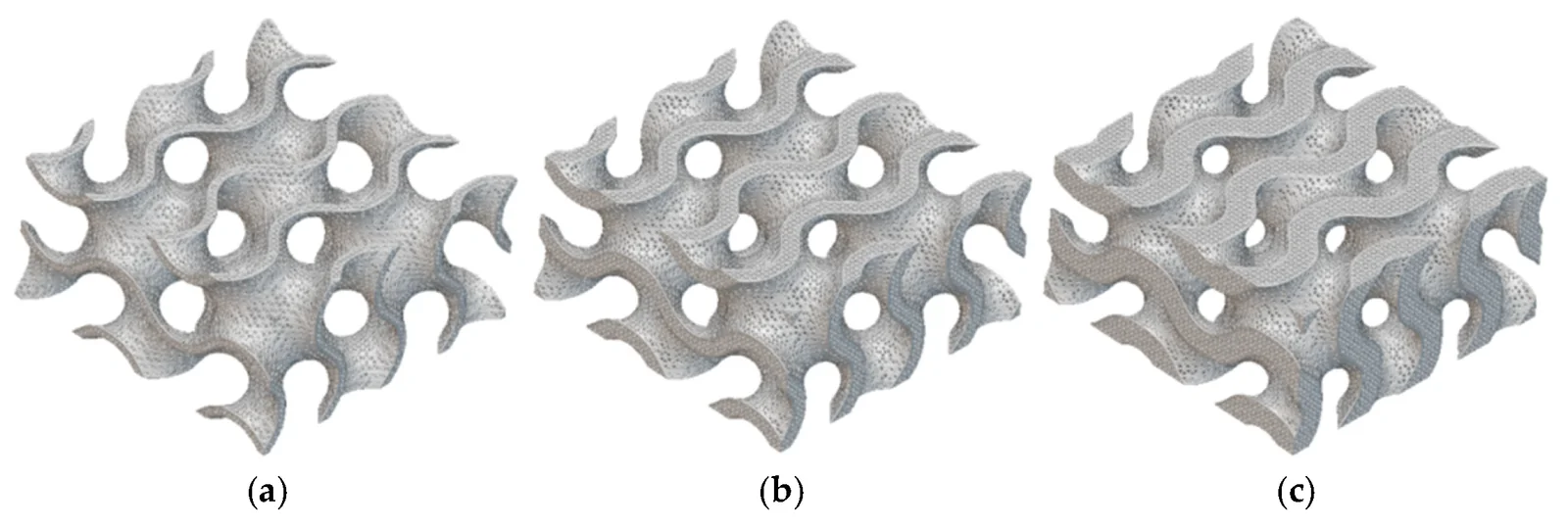
Gyroid TPMS models with relative densities of: (**a**) 15%, (**b**) 30%, and (**c**) 45%.

The gyroid TPMS lattice was generated using MSLattice with relative densities (RDs) of 15%, 30%, and 45%, maintaining common dimensions of 30 × 30 × 15 [mm], to evaluate the effects of RD on thermal performance, as shown in Figure 15. The simulations were conducted under a constant porosity of 0.8, inlet velocity of 5 [cm/s] and heat load of 60 [W] to enable consistent comparison.

**Figure 16:**
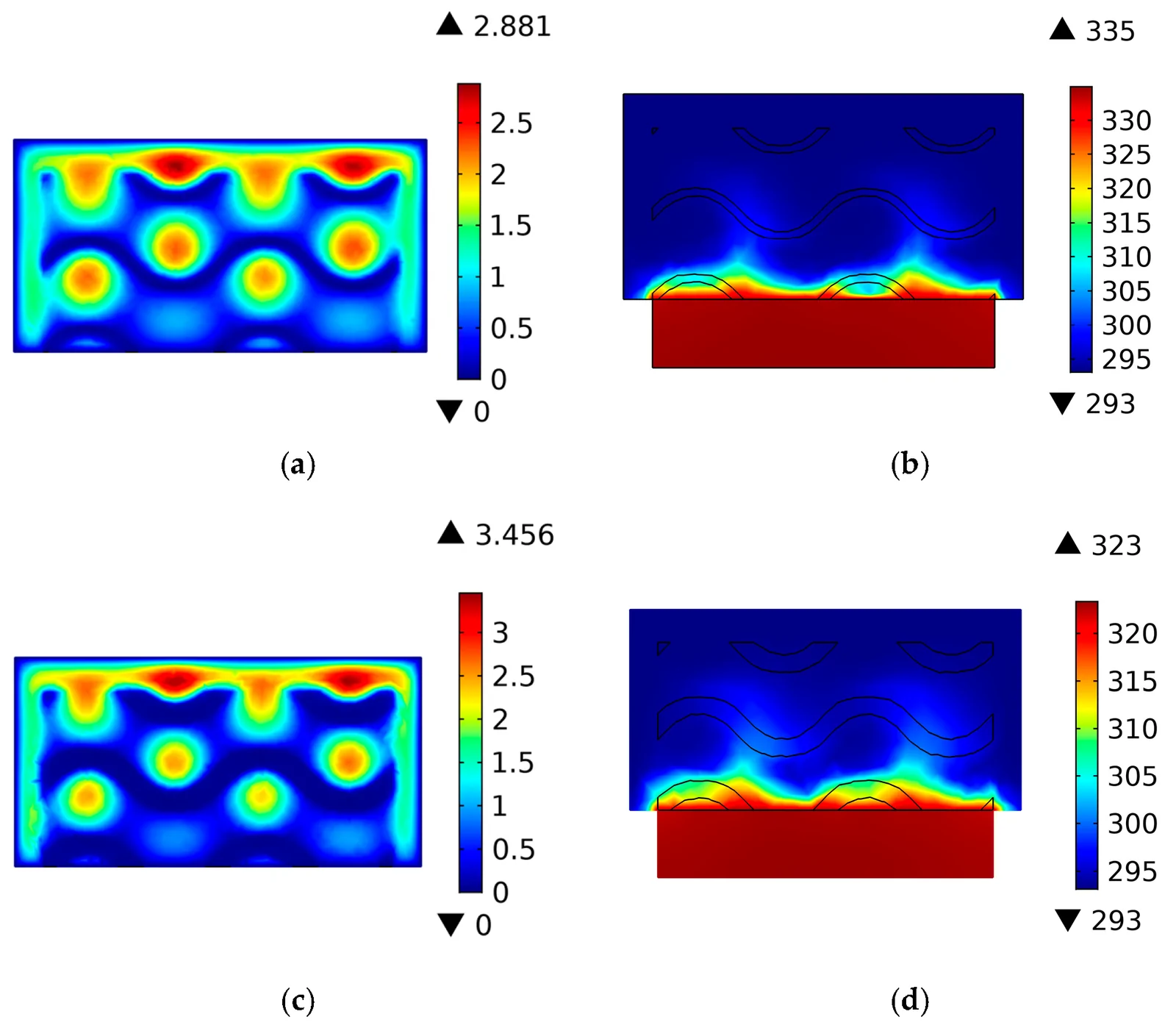

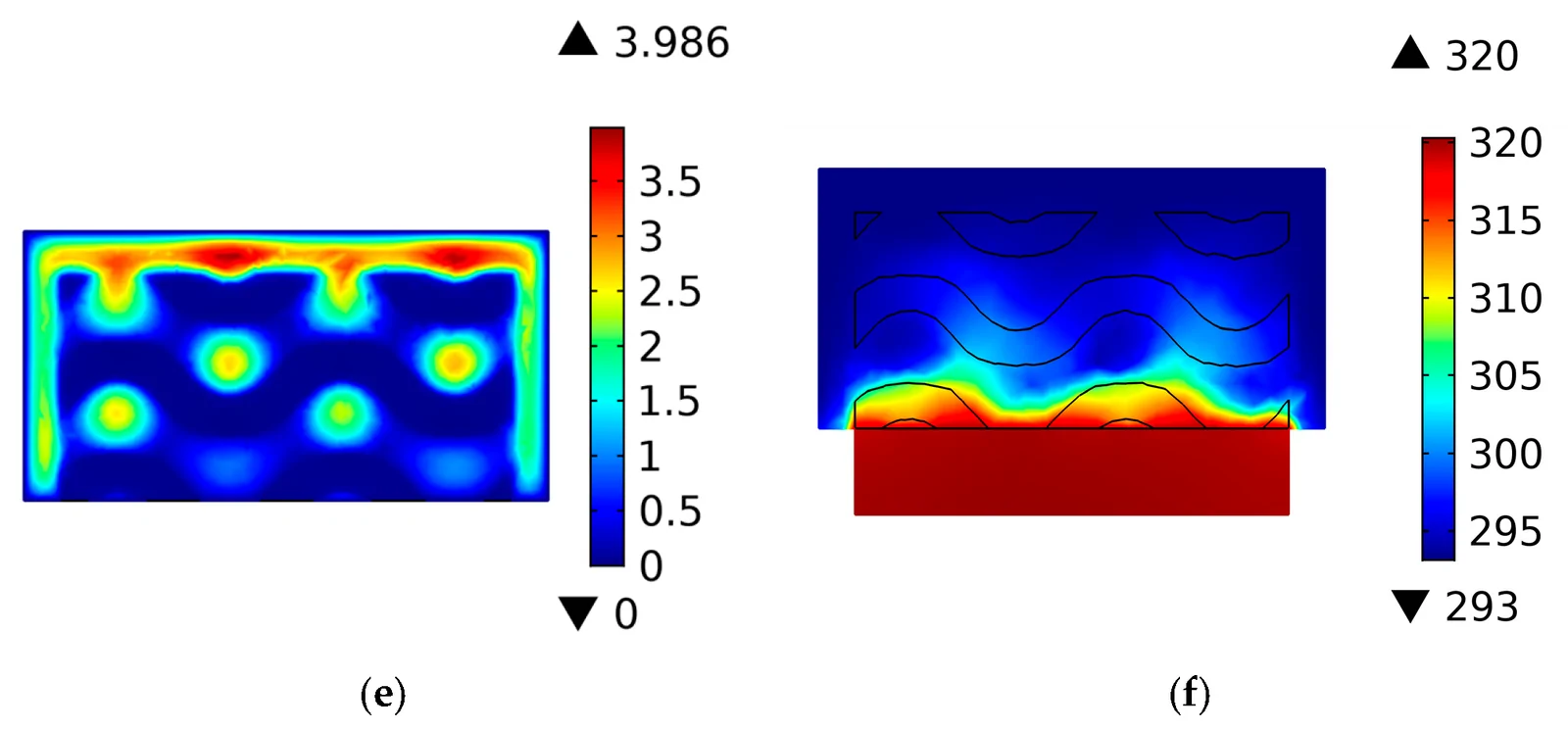
Velocity contour and temperature field for variation RD: (**a**) velocity profile at RD15%, (**b**) temperature at RD15%, (**c**) velocity profile at RD30%, (**d**) temperature at RD30%, (**e**) velocity profile at RD45%, and (**f**) temperature at RD45%.

Figure 16a,c,e illustrate the effects of RD on the velocity profile at the central cross-sectional plane of the channel. The velocity contours indicate that increasing RD from 15% to 45% progressively reduced the effective flow passage area within the gyroid TPMS heat sink. As the solid volume fraction increased, the pore throats became narrower, forcing the coolant to flow through more constricted pathways. Consequently, the fluid accelerated within these restricted regions, resulting in an increase in the maximum velocity from 2.88 [cm/s] at 15% RD to 3.99 [cm/s] at 45% RD. Overall, the maximum velocity increased by 38.5% as the RD value increased from 15% to 45%.

Figure 16b,d,f present the temperature field at the central cross-sectional plane of the gyroid structure for different relative densities. The findings show that the maximum temperature decreased from 335 to 320 [K] as the RD increased from 15% to 45%, corresponding to a temperature reduction of approximately 4.5%. This reduction can be attributed to the increased solid fraction associated with higher RD, which provides a larger heat-transfer surface area for fluid–solid thermal interaction. The enhanced surface area promotes more effective heat dissipation from the heat source, thereby improving the thermal performance of the heat sink despite the increased flow resistance. However, the overall performance of the porous TPMS heat sink depends on simultaneously maintaining low flow resistance and high heat transfer efficiency. Therefore, an optimal design should achieve a balance between hydraulic and thermal dissipation efficiency.

**Figure 17:**
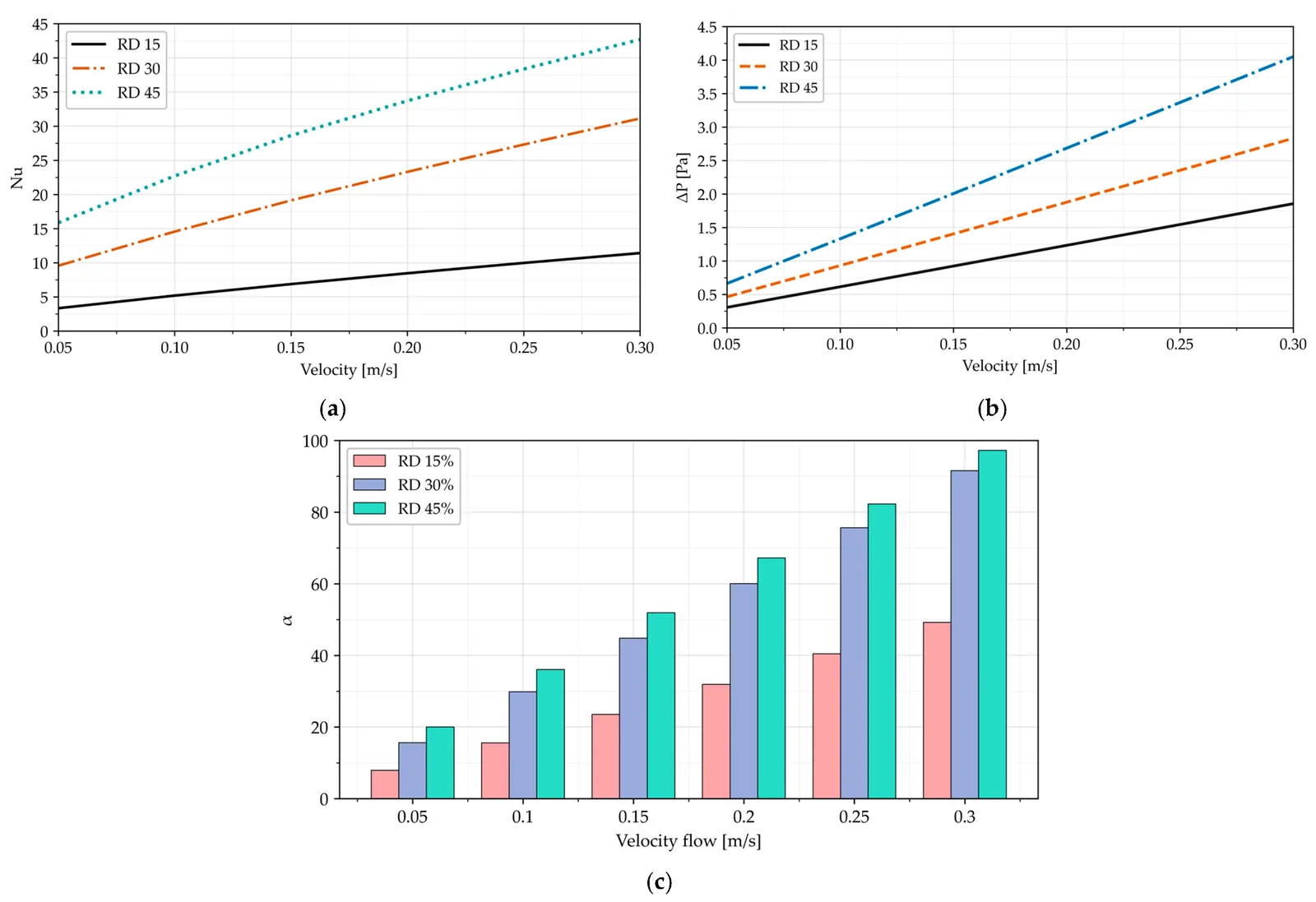
Variation in Nusselt number (**a**), pressure drop (**b**) and performance evaluation criterion (**c**) vs. inlet velocity.

The Nusselt number curves as a function of the coolant fluid flow at RDs of 15%, 30%, and 45% are illustrated in Figure 17a. To evaluate the effect of flow velocity on thermal performance, inlet velocities ranging from 0.05 to 0.3 [m/s] at step of 0.05 [m/s] were used in the numerical simulations. The results show that the Nusselt number increased with higher inlet velocity for all models. This confirms that the higher inlet velocity promoted stronger fluid mixing and enabled heat to be carried away more effectively from the source, thereby enhancing the convective heat transfer coefficient. Moreover, an effect of RD on thermal performance was observed. At a given inlet velocity, the Nusselt number grew with increasing RD. For example, at an inlet velocity of 0.3 [m/s], the Nusselt number reached 43 for RD = 45%, 31 for RD = 30% and 11 for RD = 15%. The thicker wall thickness (higher relative density) provided a larger solid surface area. This led to improved thermal performance of the structure. However, excessively thick cells reduce the fluid contact area, resulting in decreased heat transfer. In the same way excessively thin cells increase volume and permeability but decrease structural strength. Therefore, the optimal cell thickness is important in balancing heat transfer and fluid flow conditions.

The plot shown in Figure 17b presents the pressure drop versus inlet velocity for different RDs. At every inlet velocity, the RD = 45% structure exhibited the highest pressure drop, followed by RD = 30% and RD = 15%. At an inlet velocity of 0.30 [m/s], the pressure drop reached approximately 4.0 Pa for RD = 45%, compared with 2.8 [Pa] for RD = 30% and 1.9 [Pa] for RD = 15%. The greater relative density led to higher pressure losses due to reduced pore size and flow cross-sectional area; the resulting narrower and more tortuous flow paths enhanced viscous friction, thereby requiring greater pumping power to sustain a constant inlet velocity. The performance evaluation criterion (α) as a function of inlet velocity is shown in Figure 17c. The results demonstrate that the model with RD = 45% achieved a higher α compared to RD = 15% and RD = 30%. For example, at a higher inlet velocity of 0.3 [m/s], increasing the RD from 15% to 30% enhanced the α value by 85.7%. However, a further increase in the RD from 30% to 45% resulted in only a 5.5% improvement, suggesting diminishing returns due to the increased hydraulic resistance associated with higher relative densities. Generally, the results of the work reveal that the TPMS topology and the relative density of the structure are the key design parameters that determine the overall thermo-hydraulic performance of the TPMS heat sink.

## 4. Discussion

The present results demonstrate the combined effects of TPMS topology, network morphology, relative density, and inlet velocity on the thermo-hydraulic performance of a heat sink. Comparison of the temperature distribution along the channel centerline (Figure 6) shows that the gyroid lattice consistently produced the lowest and most uniform temperature field, whereas I-WP exhibited the highest localized temperature and least efficient heat removal. The diamond showed intermediate temperature results between the gyroid and I-WP. This behavior is consistent with the results of Tang et al. [17], who evaluated the convective heat transfer performance of solid TPMS lattices relative to conventional fin structures and reported that the diamond outperformed the gyroid and I-WP. This is in agreement with the ranking observed here, despite the present work considering porous rather than solid TPMS networks. The curvature discontinuities inherent to the I-WP surface promote flow separation, degrading local heat removal, whereas the diamond’s geometry promotes extreme localized cooling and the gyroid’s continuous topology promotes global temperature uniformity.

The pressure drop behavior (Figure 9) is likewise strongly dependent on lattice topology. The diamond lattice showed the highest average pressure drop, as its highly interconnected pore structure imposes substantial fluid resistance, while the continuous surface morphology of the gyroid enables smoother fluid transport and more efficient pressure recovery. The resulting ranking (diamond > I-WP > gyroid) agrees with topology-dependent trends reported in the literature. Sun et al. [22] attributed the elevated pressure drop in diamond geometries to a curvature focusing effect that intensifies local flow acceleration and wall shear near the heat source. Meanwhile, the moderate but disturbed pressure profile obtained for the I-WP in the present work is consistent with the flow separation induced by its curvature discontinuities. The smoother pressure gradient obtained for the gyroid further supports the view that pore connectivity and surface curvature rather than relative density alone govern pressure-drop behavior in TPMS heat sinks.

Regarding heat transfer efficiency, the gyroid structure achieved an average Nusselt number 10.9–12.4% higher than the I-WP, while the diamond showed a 2.9–4.7% improvement over the I-WP. This ranking diverges from the results of Tang et al. [17], who reported that the diamond achieved the highest Nusselt number among TPMS topologies under air cooling with a constant-temperature base. The discrepancy in the leading topology (diamond versus gyroid) likely reflects the choice of working fluid: air-cooled convection is dominated by boundary-layer effects at the solid surface, promoting the diamond’s uniform pore distribution, whereas water cooling, owing to its higher thermal conductivity and volumetric heat capacity promotes the gyroid’s interconnected channel network, which enhances heat transfer through secondary flow and mixing rather than surface area alone. This suggests that the optimal TPMS topology is not universal but depends on the dominant heat transfer mechanism set by the working fluid and flow regime.

The observed increase in the Nusselt number with relative density (equivalently, its decrease with porosity) is consistent with the results of Sun et al. [22], who reported that lower porosity increased the surface area and enhanced the thermal performance, and with the results of Cheng et al. [23], who found that increasing porosity reduced the heat transfer coefficient due to lower internal flow velocities. However, Cheng et al. [23] also noted that the Nusselt number can increase with porosity in certain regimes owing to characteristic length-scale effects, a trend not observed over the relative density. This difference likely arises because the present RD variation was applied to a single gyroid topology at fixed inlet velocity and channel dimensions, whereas Cheng et al. [23] compared multiple TPMS networks. The finding confirms that lattice topology rather than relative density alone determines the thermal hydraulic trade-off in TPMS heat sinks, with the gyroid offering the most balanced performance and the diamond promoting localized cooling at the expense of higher pressure drops.

## 5. Conclusions

Triply periodic minimal surface (TPMS) structures have recently attracted significant attention for thermal management applications due to their high specific surface area, interconnected flow passages, and compact design, which collectively enhance heat transfer and efficient coolant distribution. The current work presents two novel approaches. In the first approach, a numerical investigation was conducted to evaluate the influence of different TPMS types, namely the diamond, gyroid, and I-WP, on cooling performance and flow behavior by modeling these structures as porous media. In the second approach, the influence of design parameters, including lattice network type, relative density and inlet velocity, on the overall thermal-hydraulic performance of the heat sink was systematically investigated. The CFD simulations were conducted using COMSOL Multiphysics version 5.1. In the analysis, each TPMS aluminum matrix was coupled to a copper base plate subjected to a constant heat load of 60 [W]. Water was employed as the coolant in the analysis. The key findings are summarized below.

The gyroid topology demonstrated the most balanced thermo-hydraulic performance, achieving a 10.9–12.4% higher average Nusselt number than the I-WP alongside a lower pressure drop than the diamond. In contrast, the diamond yielded only a 2.9–4.7% Nusselt number enhancement over the I-WP while incurring the highest pressure drop among the three topologies. The I-WP topology exhibited the least favorable heat transfer characteristics under the investigated conditions.

The porous gyroid structure demonstrated a 2.2 [K] reduction in maximum temperature relative to the solid gyroid counterpart, indicating that the interconnected pore network enhances coolant penetration and yields measurable cooling improvements not captured by fully resolved solid-network representations. Likewise, the sheet-based gyroid structure outperformed the corresponding solid network by providing a larger effective heat transfer area and more uniform temperature distribution.

Increasing the relative density from 15% to 45% reduced the maximum surface temperature by 15 [K] (from 335 [K] to 320 [K]) and increased the average Nusselt number by approximately fourfold, whereas the pressure drop rose by only a factor of ~2.1 over the same range. Accordingly, optimal TPMS design should balance enhanced heat transfer against acceptable pumping power requirements.

The Nusselt number increased monotonically with inlet velocity across all three topologies, confirming that higher flow rates enhance coolant mixing and convective heat transfer regardless of lattice topology. Overall, these findings demonstrate that the thermo-hydraulic performance of TPMS heat sinks depends on the coupled effects of topology, relative density, structural representation, and network morphology. Therefore, these parameters should be considered together in designing future TPMS heat sinks for thermal management applications.

Experimental studies are recommended in future work to validate these findings and further characterize the heat transfer performance of the TPMS heat sink models investigated in this study.

## Figures and Tables

**Table 1:** Implicit mathematical equations and unit cell geometries of TPMS.

TPMS Lattice	Governing Equation	Iso-Surface for c = 0
Gyroid	sin(2απx)cos(2βπy) + sin(2βπy)cos(2γπz) + sin(2γπz)cos(2απx) = c	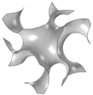
Diamond	cos(2απx)cos(2βπy)cos(2γπz) − sin(2απx)sin(2βπy)sin(2γπz) = c	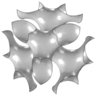
I-WP	2(cos(2απx)cos(2βπy) + cos(2βπy)cos(2γπz) + cos(2γπz)cos(2απx)) - (cos2(2απx) + cos2(2βπy) + cos2(2γπz)) = c	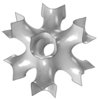

**Table 2:** Thermo-physical properties of materials used in the simulations.

Material	Density [kg/m^3^]	Dynamic Viscosity [kg/(m s)]	Specific Heat Capacity [J/(kg K)]	Thermal Conductivity [W/(m K)]
Copper	8960	-	385	400
Aluminum	2700	-	900	238
Water	997	0.89 × 10^−3^	4182	0.6

## Data Availability

The original contributions presented in this study are included in the article. Further inquiries can be directed to the corresponding authors.
